# CRIMALDDI: a prioritized research agenda to expedite the discovery of new anti-malarial drugs

**DOI:** 10.1186/1475-2875-12-395

**Published:** 2013-11-05

**Authors:** Steve A Ward, Ian C Boulton

**Affiliations:** 1 Liverpool School of Tropical Medicine, Pembroke Place, Liverpool L3 5QA, UK; 2 TropMed Pharma Consulting Ltd, 10 Brampton Chase, Lower Shiplake, Oxfordshire RG9 3BX, UK

**Keywords:** CRIMALDDI, Malaria, Drug discovery, Research agenda

## Abstract

The CRIMALDDI Consortium has been a three-year project funded by the EU Framework Seven Programme. It aimed to develop a prioritized set of recommendations to speed up anti-malarial drug discovery research and contribute to the setting of the global research agenda. It has attempted to align thinking on the high priority issues and then to develop action plans and strategies to address these issues. Through a series of facilitated and interactive workshops, it has concluded that these priorities can be grouped under five key themes: attacking artemisinin resistance; creating and sharing community resources; delivering enabling technologies; exploiting high throughput screening hits quickly; and, identifying novel targets. Recommendations have been prioritized into one of four levels: quick wins; removing key roadblocks to future progress; speeding-up drug discovery; and, nice to have (but not essential). Use of this prioritization allows efforts and resources to be focused on the lines of work that will contribute most to expediting anti-malarial drug discovery. Estimates of the time and finances required to implement the recommendations have also been made, along with indications of when recommendations within each theme will make an impact. All of this has been collected into an indicative roadmap that, it is hoped, will guide decisions about the direction and focus of European anti-malarial drug discovery research and contribute to the setting of the global research agenda.

## Background

Malaria remains a major cause of mortality and morbidity throughout the developing world, especially in sub-Saharan Africa, but also in India, Latin America, and Southeast Asia. In 2010, there were still 216 million cases and 655,000 deaths attributed to the disease [1]. It is now well established as a key challenge for the global community and a focus for international development, health funding, and assistance. Starting with the launch of the Roll Back Malaria Partnership in 1998 [2], there has been increasing recognition of the unacceptable social and economic impacts malaria makes on affected countries, as well as the suffering it brings to patients and their families. The Abuja Declaration of 2000 [3] underlined the commitment of African governments to work towards significantly reducing the burden in sub-Saharan Africa and the need for the world to reprioritize it after a relative lack of interest following the ending of the Global Malaria Eradication Programme (GMEP) in 1969. Significant resources are now channelled towards malaria control through agencies such as the Global Fund to fight AIDS, Tuberculosis and Malaria, the US President’s Malaria Initiative, the World Bank Global Strategy and Booster Program, and many others. However the development of new technologies and the past successes of GMEP in eliminating malaria from Europe, North America, the Caribbean, and parts of Asia and South-Central America have now opened up the possibility of once again seeking to eliminate and ultimately eradicate malaria globally [4]. The Malaria Forum in October 2007 (convened by the Bill and Melinda Gates Foundation) issued the challenge to the world to move from a control and containment strategy to embrace the ultimate goal of eventually eradicating the disease [5]. This was rapidly embraced by the World Health Organization (WHO), the Roll Back Malaria Partnership (RBM), and many other organizations and institutions. The development of the Global Malaria Action Plan (GMAP) in 2008 gave a roadmap for the initial stages of this long-term process [6].

The GMAP emphasized the need for a proper research and development agenda to develop the new tools which would be required to meet all the challenges. This led to the establishment of the MalERA Initiative – a two-year programme to develop a research agenda across all the disciplines and approaches relevant to tackling the disease. A recent series of papers has detailed the findings of this major collaborative project [7]. The importance of drug treatment to meeting the elimination (and ultimately eradication) challenge has been recognized from the outset.

## CRIMALDDI consortium

The CRIMALDDI Consortium was established in 2008 under a European Union Seventh Framework Programme Coordination grant. It has been a three-year project to develop a coordinated action plan for future research programmes and funding opportunities to inform the European anti-malarial drug research agenda for the next decade and also contribute to the discussions on setting the global agenda. As such it complements initiatives such as MalERA by developing more detailed plans for the high priority challenges that need to be addressed in the next five to ten years if the community is to be able to develop the drugs it needs for malaria elimination and eradication. It also focuses on the priorities that need to be addressed in the next five years as opposed to the MalERA Drug Research Agenda’s focus on drugs that will be needed specifically for eradication [8]. An earlier paper has outlined the work of the Consortium and its methodology [9].

The Consortium members identified five priority work streams to focus attention on. The approach of each work stream was to bring together global experts in a series of one- or two-day facilitated and interactive workshops to address the work stream topic. The participants in these workshops were global experts both within the field of malaria and from related fields. The introduction of experts from outside malaria was to try and bring new thinking to the topics under discussion. The five topics were:-

•Artemisinin resistance;

•Managing the wealth of new high throughput screening (HTS) data;

•*Plasmodium falciparum* and *Plasmodium vivax* novel targets and classes;

•Stage-specific screening methods;

•Using chemistry to understand biology.

The workshops were run between March and October 2010 and reports of the discussions can be found on the CRIMALDDI website [10] and in the Additional file 1, Additional file 2, Additional file 3, Additional file 4 and Additional file 5. The recommendations of the workshops and of the Consortium were periodically reviewed by an Expert Advisory Group (EAG). Reports of the EAG meetings can also be found on the CRIMALDDI website and in the Additional file 6, Additional file 7, Additional file 8 and Additional file 9. The EAG reviewed the recommendations according to three agreed parameters:

•**Progress** of the Consortium against the objectives established at the outset of the Project and agreed with the European Commission;

•**Coordination** of the outputs and recommendations with other malaria research and development initiatives;

•**Alignment** to ensure that adequate attention has been given by the workshops to the drug discovery process.

## Five key themes

Inevitably there was some overlap in the recommendations of the five workshops, but it became clear that the findings could be grouped under five key themes:

•Attacking artemisinin resistance;

•Creating and sharing community resources;

•Delivering enabling technologies;

•Exploiting HTS hits quickly;

•Identifying novel targets.

Each of these priority themes is important in moving drug discovery forward quickly to ensure that there is a portfolio of new products available to fill the demands of malaria control and elimination as well as guarding against the loss of any of the currently available anti-malarial drug products. These are summarized in Table 1.

**Table 1:** Five key themes

**Key theme**	**Rationale behind the theme**
**Attacking artemisinin resistance**	Artemisinin-based combination therapy is fundamental to the control of malaria. The appearance of tolerance to artemisinins seen in Southeast Asia could threaten current control and elimination efforts. Understanding the mechanism of resistance is fundamental to designing future anti-malarials.
**Creating and sharing community resources**	Improved sharing of information about all aspects of anti-malarial drug discovery will help to speed up drug development. Strategies have been identified to improve information sharing in order to focus effort and reduce duplication.
**Delivering enabling technologies**	Anti-malarial drug discovery is being held up, especially for *P. vivax*, because certain enabling technologies are not in place. The development of these will be the key to discovering novel drugs.
**Exploiting HTS hits quickly**	The structures of about 20,000 compounds that have given positive hits in HTS are now publicly available. Recommendations are made to filter this unparalleled amount of information quickly and efficiently to identify the most promising leads and move them quickly into drug development.
**Identifying novel targets**	At present drug development is focused on a few well-characterized drug targets, nearly all in the blood stage of *P. falciparum* infections. The search for new drug targets is constrained by the poor understanding of the underlying biology of the parasite’s life cycle in humans. New targets need to be identified and validated.

### Attacking artemisinin resistance

Artemisinin derivatives and artemisinin-containing combination therapy are now central to the first-line treatment of *P. falciparum* malaria. The recent emergence of decreased sensitivity of the parasite to artemisinins in Cambodia is of grave concern and puts at risk the entire strategy for the treatment of malaria [11]. Considerable effort is being put into the discovery of anti-malarials, which have similar modes of action to the current artemisinin derivatives or contain pharmacophores similar to the endoperoxide bridge. Examples of this type of work are the synthetic endoperoxide OZ439, sponsored by Medicines for Malaria Venture (MMV) [12], and investigations underway at the University of Liverpool into tetraoxanes as anti-malarials [13]. It is crucial to the future of this work that an understanding of the mechanism of artemisinin resistance and the degree of cross-resistance to other chemotypes, including the endoperoxide moiety, is developed urgently. The answer to this question will either close off the endoperoxides as potential future drugs or redirect work on novel structures retaining this functional group. For these reasons, developing an understanding of the mechanism of resistance and its implications for drug discovery was prioritized.

### Exploiting high throughput screening hits quickly

The publication of the structures of 13,500 compounds with high levels of activity in high-throughput primary screens against *P. falciparum* by GlaxoSmithKline (GSK) [14] and the St Jude’s Group [15] has given the malaria community a very large resource to tap into. These structures (together with more recent additions from other library screens) are publicly available through the ChEMBL [16] and Collaborative Drug Discovery [17] databases. In total there are about 20–25,000 anti-malarial “hit” structures available in publicly accessible databases. Drug discovery groups can use these to find new drug types and take them forward towards full drug development. However, only a few tens of these positive hits can realistically be progressed to full drug development. Through logical and properly validated processes, the structures must be filtered down to a manageable number. The community needs to have mechanisms for ensuring that the duplication of work is minimized and that this unique resource’s value is maximized. This in turn requires the global anti-malarial drug discovery community, as far as possible, to be aware of who is working on what within the community to ensure the timely sharing of information.

One of the problems identified from the publication of the active structures by GSK, Novartis and St Jude’s was how the community would be able to access quantities of the chemical compounds in order to carry out further work. The need for medicinal chemistry resources to make these compounds more readily available in a structured and monitored way was highlighted. MMV has subsequently responded to this challenge in part through the collation of the “Malaria Box” of some 400 chemical hits that are representative of the drug-like and probe-like molecules form the full collection. This physical set of compounds is available for researchers free of charge through MMV [18] and should serve as a catalyst to ongoing discovery research.

### Identifying novel targets

Since the publication of the malaria genome, there have been numerous efforts aimed at identifying and validating novel drug targets. Despite these efforts, the reality is that much anti-malarial drug discovery is still focused on a limited number of historic targets (the folate and haemoglobin degradation pathways are prime examples), and primarily on the blood stage of the parasite’s life cycle. There is presumably a wealth of untapped targets in the proteome (or rather proteomes) of the various developmental stages of the parasite that can be targeted. It is understandable that drug discoverers will be drawn to working on well characterized targets but the real advances in treatment in the past have been delivered when new targets or compound classes have been identified (as with artemisinins). In particular, the increasing awareness of the burden from *P. vivax* infections and the real paucity of drugs to treat them (especially the liver stage infections) underline the need to look more widely for novel targets [19,20]. A variety of approaches should be adopted to increase the chances of finding new targets, and many research groups will need to engage in order to bring momentum to this intellectually demanding challenge. However, if all this effort remains focused on a few existing targets and there is unproductive duplication of effort, then progress in overcoming the gaps in the current drug portfolio will be too slow.

### Creating and sharing community resources

As mentioned under “exploiting HTS hits quickly”, the malaria community may be moving forward more slowly in finding new drugs than it could. An absence of ways to collaborate and share information is contributing to this. The sharing of information about who is working on what and how successful they are (while not either compromising commercial confidentiality or academic demands for high-impact publications) would allow for better management of the limited resources and ensure that quantities of compounds that can be made available are not wasted.

The structural information now publicly available has come from the screening of specific chemical libraries. However there are a number of other types of libraries that have not yet been tapped into – especially those from the agrochemical sector. Tools to further filter the structures against knowledge of what is druggable and what is not have yet to be fully applied to these results. Not yet fully developed are the structures and processes within the malaria community to ensure that there is minimal unnecessary duplication of effort. Any research group can work in the same area as another if they think they will bring a new insight to that area, in fact competition can often drive the speed of delivery. That said, the priority should be, in times of restricted resources, to try to minimize unnecessary duplication.

### Delivering enabling technologies

Finding new anti-malarials is constrained by the lack of key enabling technologies. The current inability to culture infected hepatocytes long term and to screen for activity against hypnozoites is a major roadblock to identifying new drugs to treat recurrent *P. vivax* infections. Similarly the inability to culture and screen compounds for activity against other stages in the *Plasmodium* life cycle, especially in other species that infect humans, severely restricts the ability to look at these stages for possible new ways to attack the parasite. As the drug discovery agenda shifts away from just treating acute *P. falciparum* infections to eradicating asymptomatic reservoirs of parasites, progress will be slow if the tools to probe activity outside of the infected blood stages are not available. Focusing on the key enabling technologies that will break through the developmental roadblocks will, if successful, speed up finding new drugs to fill in the gaps needed not only to continue with the control phase of GMAP, but also the elimination phase.

### Prioritized recommendations

A lengthy “wish list” of lines of research and technologies whose development can be justified will not necessarily be of great value to the institutions funding such work. The resources that the major funding agencies are able to devote to malaria research may have peaked [21]. This is due both to the ongoing economic climate and also to calls on their resources to support research and development in other diseases. It can be difficult for these organizations to decide on where the priorities should be placed, especially if they are merely responding to research proposals and not pro-actively defining their own agenda. One of the objectives of the CRIMALDDI Project was to develop a prioritized and coordinated set of recommendations that could contribute to the debate over priority setting. By bringing together a range of expertise from across the disciplines involved in anti-malarial drug discovery and the related enabling technologies, it was possible to suggest a more focused set of recommendations that will expedite the work needed to meet the objectives of the GMAP [6]. In particular, the recommendations for drug discovery research could be focused on those programmes that will expedite the development of the drug tools needed when the current tools either lose their usefulness or do not meet the changing requirements of the elimination and eradication stages of GMAP.

The CRIMALDDI Consortium process took the recommendations developed during the interactive workshops and attempted to prioritize them according to their contribution to accelerating drug discovery. It then attempted to make a first estimate of the time and cost that will be needed to achieve the goal of each recommendation. It also indicated when the recommendations under each key theme could be expected to have an impact. This roadmap both met the objective of developing a coordinated plan to inform decisions on European Union support for anti-malarial research and also to contribute to the debate on a global agenda in response to the call from the MalERA Consultative Group on Drugs [8].

## Methods

### Prioritization

The series of interactive workshops held in 2010 produced a range of recommendations that have been described earlier. The CRIMALDDI Management Team then met at the start of 2011 to agree on how these could be clustered into the five key themes that had been identified as running through the workshop findings. Inevitably there was a degree of overlap and replication between the findings and this was eliminated. Some recommendations were merged together where it was judged that they covered much the same ground or were elements of a larger need.

Once the list of recommendations had been agreed, the team then agreed on a scheme to prioritize items. Four levels of priority were felt to be appropriate (Table 2):

**Important quick wins:** recommendations that fell under this heading would be those that could be relatively easily achieved in a short time frame (less than 12 months) and for relatively little cost. They might include holding consensus meetings, or improving access to existing resources. The implementation of such recommendations would allow other lines of work to proceed, but to complete them would require only a small amount of resource.

**Removing key roadblocks to future progress:** recommendations that fell under this heading would be those that seriously impede progress in key aspects of anti-malarial drug discovery. Without removing these roadblocks, there would be little prospect of delivering the major tools necessary to meet the pre-elimination and elimination goals developed under GMAP. Such roadblocks would require a major effort to remove them, and work on them should start as soon as possible.

**Speeding-up drug discovery:** recommendations that fall under this heading would, if implemented, ensure that anti-malarial drug discovery could be speeded up significantly. However, if they were not implemented, there are existing approaches or levels of knowledge that would allow research to continue, albeit more slowly and with more difficulty. Investing in recommendations prioritized under this heading should be considered once adequate resources have been put behind removing the key roadblocks.

**Nice to have:** the final heading under which recommendations could be grouped was those that could be judged to be of interest and would contribute to anti-malarial drug discovery or to developing key enabling technologies. However recommendations that fell under this heading were considered not to significantly hold up drug discovery if they were not implemented. Investment in these should only be considered if the recommendations under the other headings had been fully resourced.

Prioritization under each heading was achieved through weighting the potential gains of each recommendation.

**Table 2:** Prioritization of recommendations

**Important quick wins**	Activities that require only a few resources and can be achieved in a short period of time (less than one year)
**Removing key roadblocks to future progress**	Major obstacles to future programmes that must be overcome before key drug discovery programmes can progress expeditiously
**Speeding-up drug discovery**	Recommendations that will speed up progress but were not considered as roadblocks to new drug discovery for malaria control and elimination
**Nice to have**	Useful recommendations that would not significantly hold up drug discovery if they were not pursued

### Estimating costs and timing

Once the prioritization had been completed, the next step to meet the objectives of the Project was to make a first estimate of costs and timings for each recommendation. A meeting was held between members of the Management Team and Medicines for Malaria Venture to develop this. Inevitably this exercise would only be able to develop with order-of-magnitude estimates. A particular research project may be lucky and the solution found in a shorter period of time and for less cost than estimated. Alternatively it may be unlucky and require considerably longer than the estimates arrived at. There are many factors that would need to be considered in further refining this into a fully costed action plan, or to develop elements into a project proposal for a funding agency. For an initial exercise like this, it has been necessary to make reasonable assumptions and estimates to complete the exercise. However the estimates are of value in showing to the malaria community and its funders both the relative scale of resources needed to achieve this plan, and the timescale over which it may need to maintain support to ensure a reasonable chance of success.

## Prioritized recommendations

### Attacking artemisinin resistance

Table 3 shows the prioritization for this key theme. The prioritization was driven by the urgent need to generate enough of an understanding of what exactly is meant by “artemisinin resistance”. Once the necessary definitions of resistance had been agreed, as many groups as possible should be in a position to use a wide range of techniques to elucidate the mechanisms of resistance and so enable drug discoverers to direct their efforts appropriately. Given the importance of artemisinin-containing combination treatments (ACT) for the control of *P. falciparum* malaria, a process of “letting a hundred flowers bloom, one hundred schools of thought contend” [22] was agreed to be the most likely to answer this vital and pressing question in the shortest time.

**Table 3:** Priorities – attacking artemisinin resistance

**Important quick wins**	• Establish a clear definition of artemisinin “resistance”, stemming from a clinical observation of increased treatment failure and parasite clearance times. Include a broad profile of how resistance manifests itself, such as the window of parasite killing across the 48-hr erythrocytic cycle and correlation of PCT with the experimental *in vitro* parameters
	• Greatly improve access to resistant parasites in order to broaden as far as possible groups able to work on resistance
**Removing key roadblocks to future progress**	• Define the molecular and cellular basis of artemisinin-induced dormancy and develop easier to measure markers of dormancy and/or reduced susceptibility
	• Identify discriminatory phenotypes by systematic re‒evaluation all of the *in vitro* assays available
**Speeding-up drug discovery**	• Establish stable resistant parasite lines to improve access and broaden number of groups able to study resistance mechanisms
	• Identify and evaluate an appropriate range of “omic” approaches to search for discriminatory tools and markers of artemisinin resistance

### Creating and sharing community resources

Table 4 shows the prioritization for this key theme. The priority in this area is for more transparency and sharing of information, particularly data about the properties of potential new drug candidates. Given that resources in the field are not limitless, and that the community needs to ensure that unnecessary duplication of work is avoided, easy access to this information will allow better decision-making by funders and research teams on which lines of investigation to pursue.

**Table 4:** Priorities – creating and sharing community resources

**Removing key roadblocks to future progress**	• Develop and roll out a single reference database of information on compounds that are being investigated in malaria, integrate it with the various current databases on genes and metabolic pathways and ensure all are properly maintained
	• Improve communication between active research groups in malaria including sharing materials and resources and adoption of standard procedures for the *in vivo* and *in vitro* investigation of especially urgent challenges such as artemisinin resistance

### Delivering enabling technologies

It was clear from the discussions of the Consortium during the initial phases of this project that several key enabling technologies had to be developed rapidly to enable vital drug discovery research activities to progress. This was most noticeable in developing an understanding of the underlying biology of the liver stages of malaria (particularly *P. vivax*). The results of the prioritization for this key theme are shown in Table 5.

**Table 5:** Priorities – delivering enabling technologies

**Removing key roadblocks to future progress**	• Develop *in vitro* and *in vivo* culture methods, models, and assays that can be used widely and inexpensively to study *P. vivax* infections across all stages of the parasite life cycle and to screen drugs more effectively for activity
	• Elucidate the causes/biology of hypnozoite dormancy in *P. vivax* infections and so develop markers to differentiate hypnozoites from active infected hepatocytes
**Speeding-up drug discovery**	• Precisely define and develop novel methods and assays for evaluating drug activity against each stage of the parasite’s life cycle (with priority on early ring stages)
	• Develop an affordable humanized mouse model
	• Develop a standardized, robust and transferable culture system for the study of *P. falciparum* and *P. vivax* liver stages
	• Develop a robust and reliable falciparum and vivax exo-erythrocytic stage culture and assay system

### Exploiting high throughput screening hits quickly

The malaria community now has access to an unprecedented amount of structural information to inform drug discovery and candidate identification. However the prioritization was driven by the need to fully exploit this unique opportunity in a timely fashion. It was considered necessary to have in place systems and resources to ensure that this information is filtered rapidly down to a manageable number of compounds that can be taken through to full development in a reasonable period of time. Table 6 shows the prioritization for this key theme.

**Table 6:** Priorities – exploiting high throughput screening hits quickly

**Removing key roadblocks to future progress**	• Establish a single repository for compounds identified as positive screening hits and increase availability of these compound “powders” including increased medicinal chemistry resources to synthesize the compounds
**Speeding-up drug discovery**	• Continue routine screening of compound libraries and prioritization of positive hits in secondary screening. Agrochemical libraries are a particular priority
	• Emphasize development of better understanding of absorption, distribution, metabolism, and elimination (ADME) and toxicology of positive hits early in the discovery process
	• Use information on parasite resistance to chemical classes to probe underlying biological processes
	• Evaluate the speed of action and stage specificity of current HTS hits (currently 15.000‒20,000 novel chemotypes) to identify new chemotypes with similar pharmacodynamics to artemisinins. This should include evaluation against parasites with stable resistance to artemisinins (and other anti-malarials) and parasites arising from resistance “hot spots”

### Identifying novel targets

Much of the focus of anti-malarial drug discovery is still on the well-characterized targets in the blood stages of the *Plasmodium* life cycle. There are likely to be many tractable targets elsewhere in the life cycle and these will become more apparent once there is targeted research into these life cycle stages. Many of the challenges that will be encountered in the pre-elimination and elimination stages of GMAP are probably only solvable if new targets are identified and drugs targeted at them are developed. Therefore, there needs to be more focus on investigating these under-exploited aspects of the *Plasmodium* life cycle. The results of the prioritization for this key theme are shown in Table 7.

**Table 7:** Priorities – identifying novel targets

**Important quick wins**	• Better definition of what constitutes target validation to ensure that novel targets are real and practical
**Speeding-up drug discovery**	• Focus on looking for novelty in the first 12 hours of the ring stage (to ensure rapid kill of parasites as seen with artemisinins) and in the last 12 hours of the schizont stage
	• Focus on identifying targets other than haem in the blood stages of malarial infections
	• Using currently available mathematical tools and models developed in other biological fields to develop mathematical models of biological pathways in the malaria parasite to improve understanding of the underlying biology and identify possible novel targets
	• Phenotype parasite strains that are affected differently by each chemical class to identify characteristics that may be used to identify novel targets
	• Focus on increased understanding of the activity of current anti-malarials in high priority areas (e g, activity of 8-aminoquinolines in hypnozoites, effect of antibiotic pre-treatment on apicoplasts)

## Roadmap

The recommendations that were prioritized as “Nice to have” were not considered further for costing and timing. It was considered unlikely that the resources that would be available for anti-malarial drug research would stretch to include these activities at this time. However they still had some value and alternative funding streams might be available to allow them to proceed. They are shown in Table 8.

**Table 8:** Priorities – nice to have

**Delivering enabling technologies**	• Establish reporter systems for every stage of the parasite life cycle
	• Improve imaging tools for mechanisms of parasite drug resistance
	• Develop tools and reagents that have proved useful in other fields but are not yet available for malaria (antibodies, affinity tags)
	• Investigate the possibility of using a mature gametocyte and/or liver schizont assay system as a surrogate for activity against hypnozoites in primary screening for novel anti-vivax drugs
	• Develop a *P. falciparum* and *P. vivax* ookinete assay to complement the current *Plasmodium berghei* assay
	• Develop of a gametocyte motility assay
	• Develop tools to image the parasite’s response (metabolomics, proteomics, transcriptomics, structural algorithms) that have proved useful in other diseases (e g, tuberculosis) but have not yet been developed properly in malaria
	• Investigate how far one could use *Toxoplasma* as a model for *Plasmodium*
**Exploiting HTS hits quickly**	• Phenotype parasite strains affected differently by each chemical class to identify possible causes of differences in compound activity
**Identifying novel targets**	• Use available IT tools to cluster structures around identified biological activity against *Plasmodium*

“Quick wins” and “Removing key roadblocks” were judged to be of sufficient importance that they should proceed as soon as possible. It was estimated that around one year would be needed for all the resourcing to be put in place. Recommendations prioritized under “Speeding-up drug discovery” are likely to be possible as resources become available, once the other priorities have been properly funded. Therefore, since it is not possible at this stage to estimate when funding agencies will have the necessary resources to support these, the timing has only been shown in terms of duration and to show any timing dependencies.

**Figure 1:**
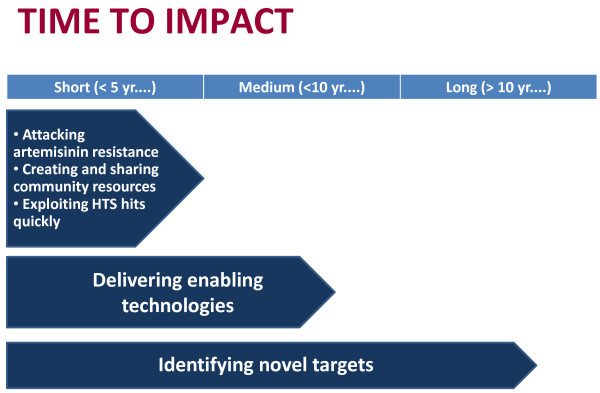
**Time to impact of key themes.** Each key theme will make an impact at a different time from when initial investment is made in it. The five key themes have been grouped according to when impact can be expected, and so can direct funders towards the themes that best match their time horizons for impact.

Different funding agencies may have different approaches to when they want to see an impact from their investment. The five key themes can be grouped according to when they are likely to have an impact on anti-malarial drug discovery. Basically, those themes that can have a discernible impact within five years of the initial investment (short term) are those that better exploit current tools. Those that can deliver an impact in ten years (medium term) are laying the foundations for future drug discovery. Finally, those that are not expected to have an impact within ten years (long term) are discovering the new tools that will be needed to meet the objectives of GMAP and malaria control, elimination and eradication. Figure 1 shows how the key themes have been grouped in this way.

**Figure 2:**
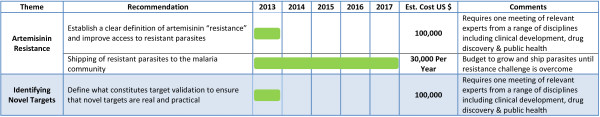
**Action plan to deliver “quick wins”.** The estimates of costs and timing needed to deliver the prioritized recommendations classified as “quick wins”.

**Figure 3:**
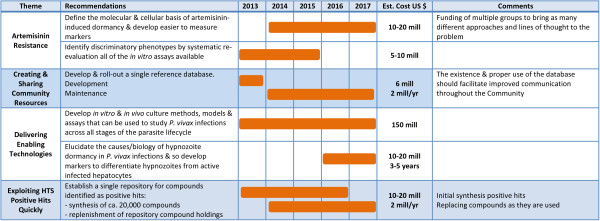
**Action plan to deliver “removing key roadblocks”.** The estimates of costs and timing needed to deliver the prioritized recommendations classified as “key roadblocks”.

**Figure 4:**
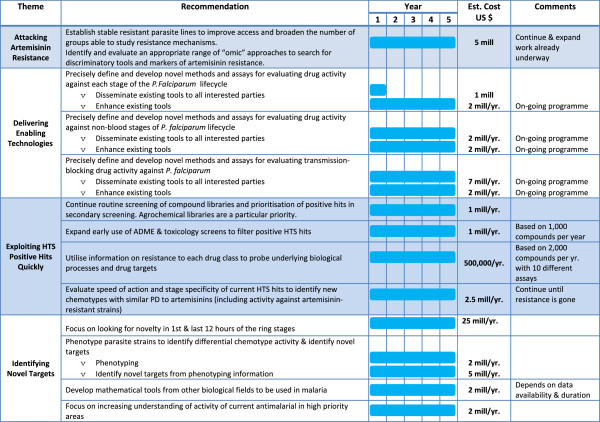
**Action plan to deliver “speeding up drug discovery”.** The estimates of costs and timing needed to deliver the prioritized recommendations classified as “speeding up drug discovery”.

Figures 2, 3 and 4 show the roadmap as developed by the process outlined earlier. They were developed by making reasonable assumptions on probabilities of success (as agreed within the CRIMALDDI Consortium).

## Conclusion

If the ambitious goal of eradicating malaria is to be achieved in the foreseeable future, then a series of new drug tools will be required to meet the new challenges. Current drug tools are adequate for control at this time and may have impact in a limited number of elimination situations. However a major research and development effort will be required to ensure that practical tools are available when needed for major eradication push in much of the malaria-endemic world (such as sub-Saharan Africa and the Indian sub-continent). To be able to focus the resources and expertise of the malaria community and its supportive funders most effectively, well coordinated plans and clear priorities are necessary. These need to be developed by the community working together so that clear and non-fragmented messages can be delivered to funders and policy-makers.

The CRIMALDDI Project, and its process of interactive workshops, has allowed the development of a set of prioritized recommendations to inform the setting of a future anti-malarial drug discovery agenda. These recommendations represent the consensus views of the participants in the workshops, drawn both from the malaria community and also drawing on expertise from outside the community, bringing in new insights to key challenges. The prioritization process and the development of a roadmap (including an estimate of cost and timing for the priorities) allows funding agencies to have a more integrated view of the strategic needs of anti-malarial drug discovery at this crucial time. It describes a coordinated action plan for European Union support for malaria research: one which can also contribute to the agenda-setting discussions among other global funding agencies. It was not the role of the CRIMALDDI project to allocate how this funding should be raised between possible funding agencies. However we hope that it will stimulate discussion and co-ordination between the agencies to maximize the use of scarce resources and to avoid unnecessary duplication of effort. The methodology of bringing together groups of experts in a particular field for interactive and facilitated workshops has proven to be a valuable way of arriving at consensus recommendations. This and the process of then strictly prioritizing recommendations, according to pre-agreed criteria, may be of value for similar initiatives in the future.

## Abbreviations

ACT: Artemisinin-containing combination treatment; CRIMALDDI: The coordination, rationalization, and integration of antimalarial drug discovery & development initiatives; EAG: Expert advisory group; EU: European Union; GMAP: Global malaria action plan; GMEP: Global malaria eradication programme; GSK: GlaxoSmithKline; HTS: High throughput screening; MalERA: Malaria eradication research agenda; MMV: Medicines for malaria venture; RBM: Roll back malaria partnership.

## Competing interests

The authors declared that they have no competing interests.

## Authors’ contributions

ICB wrote the first draft of the paper and SAW reviewed and contributed significantly to developing the final draft. Both authors have read and approved the manuscript.

## Supplementary Material

Additional file 1 **CRIMALDDI Workstream No. 1.***P. falciparum and P. vivax*: Novel Targets and Classes. Click here for file

Additional file 2 **CRIMALDDI Workstream No. 2.** Managing the Wealth of New HTS Data. Click here for file

Additional file 3 **CRIMALDDI Workstream No. 3.** Artemisinin Resistance. Click here for file

Additional file 4 **CRIMALDDI Workstream No. 4.** Stage-specific Screening Methods. Click here for file

Additional file 5 **CRIMALDDI Workstream No. 5.** Using Chemistry to Understand Biology. Click here for file

Additional file 6 CRIMALDDI Consortium: Expert Advisory Group Meeting No. 1. Click here for file

Additional file 7 CRIMALDDI Consortium: Expert Advisory Group Meeting No. 2. Click here for file

Additional file 8 CRIMALDDI Consortium: Expert Advisory Group Meeting No. 3. Click here for file

Additional file 9 CRIMALDDI Consortium: Expert Advisory Group Meeting No. 4. Click here for file
