# Supplementary material for: CRIMALDDI: a prioritized research agenda to expedite the discovery of new anti-malarial drugs
Source: Malar J. 2013 Nov 5;12:395. doi: 10.1186/1475-2875-12-395 (PMC3830512; doi:10.1186/1475-2875-12-395)
Supplement: Additional file 1 — CRIMALDDI Workstream No. 1.P. falciparum and P. vivax: Novel Targets and Classes. [file 1475-2875-12-395-S1.pdf]

CRIMALDDI CONSORTIUM

(Co-ordination, Rationalisation, & Integration of Antimalarial Drug Discovery & Development Initiatives)

EXPERT ADVISORY GROUP

MEETING No. 1

Washington 18 November 2009

Page intentionally left blank

Attendees:

CRIMALDDI Consortium	
Steve Ward (SAW)	Scientific Coordinator
Susan Jones (SJ)	Project Manager – EU Management office
Ian Boulton (ICB)	Consultant project coordinator
Expert Advisory Group	
Simon Croft	London School of Hygiene & Tropical Medicine
John Adams	University of South Florida
Ken Duncan	Bill & Melinda Gates Foundation
David Fidock	Columbia University
Chris Plowe	University of Maryland
Geoff Targett	London School of Hygiene & Tropical Medicine

Pre-meeting Reading

The document embedded in the Annex was distributed to the EAG members as preparatory reading for the meeting. The discussion at the meeting was based on this document.

Meeting Objectives

The meeting objectives were reviewed and agreed by the EAG:-

- Review process
- Review analysis to-date
- Review prioritisation of workstreams
- Review engagement with wider community
- Recommend additional aspects of malaria drug discovery to consider
- Recommend any changes to process or findings to-date

Process & Analysis To-date

The CRIMALDDI project had originated in the need for a prioritised strategy & action plan on antimalarial drug discovery that could be used as the basis for discussions on the Framework Planning round of EU funding. However it was expected that it would be of wider use to the malaria community.

The EAG were comfortable with the process and the gap analysis that had been undertaken. No significant areas or issues were identified as having been excluded.

Chris Plowe confirmed that the CRIMALDDI process supplemented & complemented the MalERA process. MalERA (for drugs) is attempting to develop an R&D agenda to address elimination leading to eradication. CRIMALDDI is looking at an R&D agenda in the nearer term and addressing issues in the Sustained Control phase of the GMAP process leading on to Elimination. Drugs that may be developed from a CRIMALDDI prioritised programme would have to be effective at low levels of malaria infections and be effective in both treating infections and be appropriate for mass drug administrations in elimination programmes.

Workstream Prioritisation

The 5 priority workstreams that had been identified (with workstream leaders) are shown in the table below:-

Workstream	Leaders
Pf novel targets & classes	Christian Doerig<br>Kelly Chibale
Pv novel targets & classes	Christian Doerig<br>Kelly Chibale
New hit-to-candidate process	Steve Ward<br>Ian Bathurst
Artemisinin resistance	Steve Ward<br>Michael Lanzer
Stage specific screening models	Donatella Taramelli<br>Henri Vial

The EAG asked to see the other workstreams that had not been prioritised in the top 5. This was not available at the meeting but would be circulated to the EAG afterwards. These other workstreams were:-

Workstream	Reason not to prioritise
Novel combinations	Would first require the identification and development of new chemotypes from which to design appropriate novel combinations
Plant derived products	Not considered readily analysable in the terms of the CRIMALDDI project.
Novel drugs for severe malaria	Need appropriate models to study. Also likely that a first step would be to identify novel chemotypes and then compounds with the appropriate PK/PD properties for severe malaria.
Novel drugs for malaria in pregnancy	Need appropriate models to study. Also likely that a first step would be to identify novel chemotypes and then compounds with the appropriate PK/PD properties for severe malaria.
Drugs for mass administration	Starting point would be novel chemotypes form which drugs with appropriate efficacy & safety profiles can be identified.
Drugs to overcome resistance mechanisms (non-artemisinin)	The development of novel chemotypes would, by definition, identify molecular frameworks that could overcome resistance by different mechanisms. A specific effort to find molecules of the same class to overcome resistance would not be the most productive way forward.

The EAG felt that the topics for the workstreams needed to be tightened up and more closely defined. The current high level descriptions ran the risk of making the workshops too unfocused and so not enabling well defined conclusions to be written. As currently written, the themes were not particularly compelling and ran

the risk of generating the same old answers to the same old questions. In particular, they recommended that the following themes be tighten up along the following lines:-

Artemisinin resistance	Identify the mechanism(s) of artemisinin resistance in order to be able to design strategies to overcome or avoid it through novel combinations or novel molecular designs that counter the mechanism(s).
Hit-to-lead process	Given the large number of molecular structures that have given positive hits in the HTS screens and which are to be release by the pharmaceutical industry (>20,000), to develop systems to:-<br>▪ Make the information available to the community in an accessible way;<br>▪ Filter the structures with robust methods to identify those structures which are druggable and more promising starts for lead optimisation;<br>▪ Allow the community to know who is working on which structures so that duplication can be avoided and resources not unnecessarily wasted.
Pf & *Pv* novel targets & classes	Develop an understanding of the underlying biology of the parasite at different stages of its lifecycle and then use this knowledge to design chemistry to act on the targets identified.

This will be addressed as a matter of urgency. The Hit-to-lead workstream needed to be renamed as the current name is potentially misleading.

The EAG felt that the workstreams on *Pf* & *Pv* novel targets & classes should be collapsed into one to allow for a new workstream. Ken Duncan recommended that there should be a further workstream to look at ways of using the results from whole cell screening to allow insights into the underlying targets of different drug types – using the chemistry to drive understanding of the biology. The output would be to identify novel targets from this understanding. This recommendation will be taken away and discussed with the rest of the Consortium.

The EAG strongly recommended that no more than 50% of the participants at the workshops should be from the malaria community. This is to avoid people simply focusing on their particular areas of interest and to get ideas in from other fields of medicine.

It was also recommended that the project should not lose sight of the potential for leveraging the platform technologies identified in the process across other neglected tropical diseases. This could be used to improve the justification for a particular priority programme in the action plan.

Engaging with the Community

The EAG endorsed the communications plan and the efforts that the Consortium was making to engage the malaria community. It was felt that it was an ambitious goal to complete the process in time for the 2010 ASTMH meeting. However the EAG agreed that this was the best meeting to publicise the results of the project and leaving it till 2011 would be too late to have any meaningful impact on either Framework Planning funding in Europe or on the funding priorities of other agencies & organisations.

Once the project has developed the workshops in detail and some output is being generated, then the CRIMALDDI team will reach out to funders (like Wellcome Trust) to engage them. Gates Foundation is part of EAG (through Ken Duncan), MMV & WHO are part of Consortium.

The EAG recommended that the Consortium publish a series of papers outlining the project, its process, and the various results of the workshops as they became available. It was pointed out that WWARN had been successful in raising its awareness in this way.

Next Meeting

It was agreed that the EAG should meet again at the end of the June / beginning of July 2010 to review the outputs of the workstream workshops and before the consolidation of the action plans had taken place. This would allow the EAG to advise the Consortium on the outputs and prioritisation. A final meeting would then be held in advance of any presentation at ASTMH 2010. Additional members from India and Africa, as well as a representative from the pharmaceutical industry, would be invited. The team would also look for possible candidates from Latin America, China, Japan, & S Korea. However the numbers would be kept below 10 to allow for good debate among the EAG members

Next Steps

Action	Responsibility	Target date
Circulate list of all workstreams that were included in the prioritisation	ICB	ASAP
Refine workshop objectives and circulate to the EAG and the CRIMALDDI consortium for comment	SAW / ICB	30-11-09
Identify possible names of participants for the workshops	EAG	31-12-09
Date of EAG meetings in 2010 to be fixed:<br>▪ Late June, early July<br>▪ Pre-ASTMH 2010	ICB / SJ	ASAP
Identify additional members of EAG to be invited:<br>▪ Africa<br>▪ Latin America (via PAHO?)<br>▪ India (SC to identify candidates)<br>▪ China<br>▪ Japan<br>▪ S Korea	ICB / SC / SAW	31-01-10
Introductory paper(s) on CRIMALDDI	SAW	ASAP
Discuss with Rob Newman (WHO GMP) to avoid any conflict with GMAP	SAW / ICB	ASAP

ICB/icb

23 November 2009.

Annex: EAG Pre-meeting Reading

The embedded document was distributed to the EAG ahead of the meeting as preparatory reading:-

CRIMALDDI EAG
Briefing 18-11-09 v1.

Chairman's Confirmation

This is to confirm that this report represents a fair summary of the discussions of the CRIMALDDI Expert Advisory Group held on 18 November 2009.

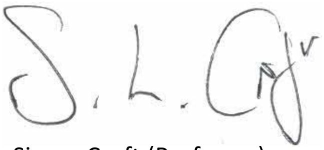

Simon Croft (Professor)
London School of Hygiene & Tropical Medicine
