# Supplementary material for: CRIMALDDI: a prioritized research agenda to expedite the discovery of new anti-malarial drugs
Source: Malar J. 2013 Nov 5;12:395. doi: 10.1186/1475-2875-12-395 (PMC3830512; doi:10.1186/1475-2875-12-395)
Supplement: Additional file 2 — CRIMALDDI Workstream No. 2. Managing the Wealth of New HTS Data. [file 1475-2875-12-395-S2.pdf]

CRIMALDDI CONSORTIUM

(Co-ordination, Rationalisation, & Integration of Antimalarial Drug Discovery & Development Initiatives)

EXPERT ADVISORY GROUP

MEETING No. 2

London 07 September 2010

Page intentionally left blank

Attendees:

CRIMALDDI Consortium

Ian Boulton (ICB)	Consultant project coordinator

Expert Advisory Group

Prof Simon Croft	London School of Hygiene & Tropical Medicine
Prof John Adams	University of South Florida
Dr Federico Gomez de las Heras	*formerly* GlaxoSmithKline
Dr Saman Habib	Central Drug Research Institute, Lucknow
Dr Jean-Rene Kiechel	Drugs for Neglected Diseases Initiative
Prof Dominique Mazier	INSERM
Prof Odile Mercereau-Puijalon	Institut Pasteur
Prof Geoffrey Targett	London School of Hygiene & Tropical Medicine

Meeting Objectives

The meeting objectives were discussed and agreed by the EAG:-

- Review and comment on progress of the project against its objectives
- Ensure the co-ordination of the project's process with the other major players
- Review the project's attention to the actual drug discovery and development process

Review of EAG Terms of Reference

The EAG reviewed its terms of reference (ToRs) as agreed at the first meeting in Washington (Nov 2009). These are:-

1. Review proposals for priorities and action plans in drug discovery
2. Advise on priorities not identified but needed
3. Advise on research groups not identified by Consortium
4. Endorse the final priorities and action plan
5. Ensure no conflict with GMAP, and advise on resolution of any conflicts

As there were several members of the EAG who had not attended the Washington meeting, some clarifications were sought. It was agreed that the Washington meeting had discharged Item 2 in the ToRs and the workstream priorities could not be changed. It was also understood that the term GMAP in Item 5 also incorporated other related initiatives (such as MalERA). Members of the EAG were worried that the CRIMALDDI initiative should not lead to more fragmentation of the thinking about the long-term direction of antimalarial drug R&D. The EAG was reminded that Prof Plowe had, in Washington, reassured the group that there was no inherent conflict between the long-range thinking that was undertaken by MalERA's Drug Discovery Workstream and the more short- to medium-term horizon of CRIMALDDI. The two initiatives are complementary.

Some EAG members questioned the lack of reference to eradication in the work of CRIMALDDI. It was pointed out that the time horizon for CRIMALDDI made thinking about eradication alone outside the current scope of the initiative. It was also noted that the tools needed for eradication would not (at least initially) be different from those needed to eliminate the disease at a country or regional level.

Review of Workshops

The EAG noted that its role was not one normally undertaken by an expert group as it was not reviewing the science of specific projects. Its role in this case is to ensure that overall initiative remains in line with its objectives and that the workshops are delivering on the key dimensions of CRIMALDDI. It was agreed that each workshop would be reviewed against the following headings:-

- Progress: Progress of workstream against CRIMALDDI objectives;
- Co-ordination: Co-ordination of the outputs of the workshops with other initiatives;
- Process: Ensuring that the outputs pay attention to the drug discovery process;

Workshop No 2: "Managing the wealth of new HTS data"

**Progress**
• An excellent output from a great workshop.<br>• Clearly a good first step in setting up a valuable resource for the malaria community.<br>• Next step will be a big challenge. Who will be in a position to drive this?<br>• This workshop highlights the need for greater community engagement. It is to be hoped that the funding agencies will be able to help facilitate this.<br>• There is a clear need for experimental collaboration to follow on from the collaboration on the use of the HTS information (*e.g.* on PK, ADME, drug-drug interactions). This could be an area where the EU could have a role that is not yet filled by any other funding body.
**Co-ordination**
• EAG felt that, although MMV´s contribution to this process is important, more thought needs to be given to the question of whether MMV is the best organisation to co-ordinate efforts in this area.<br>• There is a clear need for proper assay validation and the EAG recommended looking at the NCCLS (now CLSI) model for ensuring good cross-validation of assays.<br>• The EAG noted that the Indian Council of Scientific and Industrial Research has set up the Open Source Drug Discovery (OSDD) Initiative (see www.osdd.net) and this is now looking at malaria following on from TB work. This may be a pre-existing framework for the type of collaborative reference database recommended by the workshop. CRIMALDDI might like to be present at the next OSDD meeting which will be on malaria.
**Process**
• The workshop has made the necessary links to the drug discovery process. Links to essential next stages (like ADME, PK, drug-drug interactions, *etc.*) have been made.<br>• The EAG noted that the use of Lipinski rules to assess druggability is often not useful in anti-infectives.

Overall the EAG was very happy with the output from this workshop.

Workshop No 5: "Using chemistry to understand biology"

**Progress**
• It was agreed that the workshop had completed the analysis of the situation and had identified possible routes to achieve the goal outlined in the workshop question.<br>• However the EAG felt that the workshop's recommendations were too broad and did not lend themselves easily to the development of an action plan that would be of use to CRIMALDDI's ultimate audience.<br>• The EAG felt that the workshop would have been better to have stuck to the original question and not reformulated it. The new question lacked the focus that the workshop needed. The workshop needed to be clearer on the balance between obtaining short-term results using existing techniques and the longer-term objective of developing new techniques and tools (like various -omics) that would yield valuable information sometime in the future.<br>• The EAG felt that the recommendation to use drug resistance as a tool to understand a drug class' mode of action was misleading. For example, chloroquine resistance bears no relationship to the mode of action of the drug against the parasite.
**Co-ordination**
• The EAG could not discern any attempt in the recommendations to co-ordinate these recommendations with other similar agenda-setting initiatives. However it was noted that this may well be due to the lack of attention to this aspect of agenda setting by other groups.
**Process**
• The EAG felt that there was poor linkage between the recommendations and the drug discovery process. However it also noted that until many of the tools recommended by the workshop could be developed (longer-term objective), it would not be easy to make such a linkage.

Workshop No 4: "Stage specific screening methods"

**Progress**
• The EAG welcomed the focus on the need to develop new tools to study *P vivax* and liver stage infections. This was felt to be a real priority given the importance of vivax to the elimination of malaria.<br>• The EAG disagreed with the conclusion of the workshop that there was no need to screen for vivax activity in a primary blood stage screen. This conclusion was based on the fact that all drugs active against falciparum in such a screen had subsequently been found to be active against vivax blood stage. However the EAG noted that since there was no primary screen for vivax, many compounds with activity against vivax but not falciparum may be rejected in a HTS campaign. Thus they would never get to be tested in a vivax screen.<br>• The EAG again noted that there was an inherent tension between the short-term need to generate results with existing tools and the longer-term need to develop new tools. The EAG would have liked to see short-term recommendations about practical alternatives where the main recommendations are (of necessity) long-term.<br>• The EAG questioned the need to include screens for activity against the sexual stages in the mosquito. It was felt that any practical drug would only need to block gametocytogenesis in the human host. Drugs that only acted against the mosquito stages would be impractical for disease control and elimination. The EAG felt that there needed to be more clarity about the endpoint

being sought.<br>• The development of a robust mouse model for *P vivax* was a high priority but members of the EAG wondered if this was practical in a reasonable timeframe.<br>• The EAG questioned the lack of any recommendations from the workshop about identifying PK properties of compounds with *P falciparum* activity early in the screening process, especially in the animal models or screens being used.<br>• The EAG would have liked to have seen some recommendations from the workshop on the number of centres that would be needed for screening and the management of such a network.<br>• The EAG would also have liked to have seen some recommendations about ensuring the quality control and validation of the screening methods used and the centres applying them.
**Co-ordination**
• The workshop's recognition of the importance of vivax needs to be communicated and co-ordinated with some of the main funding agencies who prefer to focus on *P falciparum* research and drug discovery alone.<br>• Target product profiles (TPPs) for new products that are derived from the screens that the workshop is recommended should be aligned with the major players in developing strategy – MMV, TDR, and MalERA. This is especially true of the TPP for a sexual stage agent.
**Process**
• The workshop recognised the linkage to the various stages of the drug discovery process.<br>• However the EAG would have liked to have seen more attention to the need to look at PK, toxicology, drug-drug interactions, *etc* earlier in a screening process.

Future Workshops

Workshop No 1: "*Pf* and *Pv* Novel Targets & Classes"

The EAG noted that no-one from GlaxoSmithKline was included on the list of invitees.

Workshop No 3: "Artemisinin Resistance"

The EAG recommended that there needs to be participation from at least one clinician with direct experience of the situation in Cambodia. If Pascal Ringwald (WHO-GMP) is not able to attend, then an alternative needs to be identified.

Engaging with the Community

The EAG reviewed the community engagement plans and activities to-date. It was felt that the CRIMALDDI team was doing all it could to raise awareness and engaging the community. The EAG sympathised with the team over the lack of response. It was noted that the MalERA website also did not seem to have generated much response from the community despite its high visibility.

There is an upcoming molecular parasitology meeting that may be an opportunity to promote CRIMALDDI. John Adams to send details.

Several EAG members felt that the final report and action plan from CRIMALDDI would need to be written in a less neutral scientific style and more to "sell" the final recommendations. The style of the reports from the workshops is appropriate. However, the final report will need to communicate a level of excitement about the recommendations that is not seen in the workshop reports.

The EAG noted the planned evening event at ASTMH (04 November) and welcomed it as a first step in communicating the recommendations of the initiative. The importance of “selling” the recommendations and communicating excitement was again emphasised.

Next Meeting

The next meeting of the EAG is scheduled for Tuesday 02 November 2010 at the Marriott Marquis Hotel in Atlanta, GA (USA) in the afternoon. This will be immediately before the ASTMH Annual Meeting. Full details of the meeting will be circulated ahead of the meeting. It was also agreed that another EAG meeting at the end of the first quarter of 2011 would be required to review and endorse the final CRIMALDDI report and action plan.

The EAG were concerned about the short period of time between the last workshop (25-26 October) and the date they would want to see the report on the workshop (Fri 29 October). They were reassured that a draft report would be written in time although it would not be approved by all the workshop participants before the EAG meeting.

EAG members were asked to send agenda items to Ian Boulton as soon as possible. Two items were identified:-

- Presentation of the recommendations to communicate excitement.
- Co-ordination of the recommendations with MalERA.

Next Steps

Action	Responsibility	Target date
Report on EAG meeting drafted and circulated to members for comment and approval	ICB	ASAP
Agenda items for November EAG to be sent to Ian Boulton	All	15-10-10
Details of Molecular Parasitology Meeting to be sent to Ian Boulton	JA	ASAP
EAG pre-meeting reading to be circulated to EAG members	ICB	29-10-10
Details of the OSDD meeting on malaria to be sent to Ian Boulton to see if CRIMALDDI can participate	SH	30-09-10

ICB/icb
13 September 2010.

Chairman’s Confirmation

This is to confirm that this report represents a fair summary of the discussions of the CRIMALDDI Expert Advisory Group held on 07 September 2010.

Simon Croft 22.10.2010

Simon Croft (Professor)
London School of Hygiene & Tropical Medicine
