# Supplementary material for: CRIMALDDI: a prioritized research agenda to expedite the discovery of new anti-malarial drugs
Source: Malar J. 2013 Nov 5;12:395. doi: 10.1186/1475-2875-12-395 (PMC3830512; doi:10.1186/1475-2875-12-395)
Supplement: Additional file 3 — CRIMALDDI Workstream No. 3. Artemisinin Resistance. [file 1475-2875-12-395-S3.pdf]

CRIMALDDI CONSORTIUM

(Co-ordination, Rationalisation, & Integration of Antimalarial Drug Discovery & Development Initiatives)

EXPERT ADVISORY GROUP

MEETING No. 3

Atlanta 02 November 2010

Page intentionally left blank

Attendees:

Expert Advisory Group

Prof Simon Croft	London School of Hygiene & Tropical Medicine (Chair)
Prof John Adams	University of South Florida
Dr Ken Duncan	Bill & Melinda Gates Foundation
Dr Laurent Fraisse	sanofi aventis
Dr Jean-Rene Kiechel	Drugs for Neglected Diseases Initiative
Prof Dominique Mazier	INSERM
Prof Geoffrey Targett	London School of Hygiene & Tropical Medicine

CRIMALDDI Consortium

Prof Steve Ward (SAW)	Liverpool School of Tropical Medicine (Scientific Co-ordinator)
Prof Kelly Chibale	University of Cape Town
Dr Foluke Fakorede	TDR
Prof Donatella Taramelli	University of Milan
Prof Henri Vial	INSERM
Ian Boulton (ICB)	Consultant – Project Coordinator
Susan Jones (SJ)	Liverpool School of Tropical Medicine (EU Projects Manager)

Meeting Objectives

The meeting objectives were discussed and agreed by the EAG:-

- Review and comment on progress of the project against its objectives
- Ensure the co-ordination of the project's process with the other major players
- Review the project's attention to the actual drug discovery and development process

Review of EAG Terms of Reference

The EAG reviewed its terms of reference (ToRs) as agreed at the first meeting in Washington (Nov 2009). These are:-

1. Review proposals for priorities and action plans in drug discovery
2. Advise on priorities not identified but needed
3. Advise on research groups not identified by Consortium
4. Endorse the final priorities and action plan
5. Ensure no conflict with GMAP, and advise on resolution of any conflicts

Review of Workshops

The EAG (as it had at its second meeting in London) noted that its role was not one normally undertaken by an expert group as it was not reviewing the science of specific projects. Its role in this case is to ensure that overall initiative remains in line with its objectives and that the workshops are delivering on the key dimensions of CRIMALDDI. It was agreed that each workshop would be reviewed against the following headings:-

- <u>Progress:</u> Progress of workstream against CRIMALDDI objectives;
- <u>Co-ordination:</u> Co-ordination of the outputs of the workshops with other initiatives;
- <u>Process:</u> Ensuring that the outputs pay attention to the drug discovery process;

The EAG noted that the inter-relations between the various workshops were even clearer now than had been at its last meeting. The use of "-omics" as a way to bind together some of the themes from all the workshops was highlighted. It was noted that the use of *e.g.* metabolomics had been slow to get off the ground in TB, but was now beginning to show interesting results.

They also emphasised to the Management Team the need for a good and robust prioritisation exercise of all the recommendations from the various workshops before finalising the project's report. This would be a point that the EAG would be looking for at its next review of CRIMALDDI.

Workshop No 1: "*Pf* and *Pv* Novel Targets & Classes"

Progress

- This workshop had a large remit and could have been very unfocused. The EAG was pleased to see that it focused on 2-3 key themes.
- The report clearly delineates the recommended areas for focus. In particular, the EAG supported the clear recommendations for increased chemical diversity and the methods to achieve this. The development of a biomarker for *P vivax* hypnozoites was supported as a priority. They were unsure about the value of the *Toxoplasma gondii* model in malaria but recognised this was a controversial area.
- The EAG welcomed the priority given to the development of robust models, the need to properly validate them, and for collaboration between centres in this process. It hoped that CRIMALDDI would take a stand on the need for validation to be undertaken properly. This should be a very strong message to the EU.
- The balance between Control and Elimination phases and their different requirements was welcomed.
- The funding of the recommended databases (both from this workshop and Workshop 2) would be critical.
- The main issue is whether the recommendations would be fundable and sustainable given the grant cycles, especially in academia. In the final report, it was recommended that CRIMALDDI show where findings could achieve their objectives in the normal funding cycle and where a longer timeframe would be necessary.

Co-ordination

- The EAG emphasised the need to identify interfaces with funders and the research community to bring the recommendations to fruition. Each funder should be approached with their particular research interest in mind to gain maximum impact.
- This is a topic that needs to be discussed in depth with organisations like the Gates Foundation, NIH, Wellcome Trust

Process

- The EAG considered that the recommendations were totally relevant to the drug discovery process.

Overall the EAG was very happy with the output from this workshop.

Workshop No 3: "Artemisinin Resistance"

Progress

- The EAG welcomed the approach of starting from clinical need and working back towards the underlying biology. This needs to be emphasised more in the final report. The EAG welcomed the focus on the phenotype as a key issue to be addressed.
- The EAG would like to see more detail on timelines for the recommendations in the final report. It would be of great value if the report could show what priorities should be in 5, 10, & 15 years.
- The recommendations were a good starting point for a detailed action plan to address this important challenge. Past experience (*e.g.* development of resistance in schistosomiasis) has shown the importance of properly defining the right questions at the outset.
- The EAG shared the concerns expressed at this workshop about the need for more sharing of information, parasites, and findings.

Co-ordination

- The workshop clearly shows how important co-ordination is in this field.
- These recommendations need to be properly communicated to the sponsors of the current work being undertaken on artemisinin resistance. The outputs from the NIH meeting being held at the same time as the EAG meeting also need to be checked for alignment with the CRIMALDDI recommendations. This may need to be reflected in the final report.

Process

- The EAG felt that the recommendations interfaced well with the drug discovery process.

The EAG felt that this was an excellent meeting.

Engaging with the Community

SAW outlined the initial thinking on the preliminary presentation to be made at ASTMH. The slide set used at this evening event is embedded below:-

CRIMALDDI\ASTMH\
ASTMH Presentation

[Double-click to open embedded file]

The EAG emphasised the need to focus on the applied aspects for the funders – what needs to be done and what can be left. This helps them decide on priorities to be supported. CRIMALDDI should make clear what the main roadblocks are and how their recommendations will remove them.

Several EAG members repeated the comments made at the September meeting that the final report and action plan from CRIMALDDI would need to be written in a less neutral scientific style and more as an advocacy document to "sell" the final recommendations. The style of the reports from the workshops is appropriate. However, the final report will need to communicate a level of excitement about the recommendations that is not seen in the workshop reports. It will need to communicate clearly how CRIMALDDI is different from other initiatives (especially MalERA) and what has been its unique skill set or technique in arriving at its recommendations. It should also focus on where the EU can make a specific contribution to differentiate itself from other funders – where it can have "ownership".

Next Meeting

The EAG agreed to meet one more time to review the final report of the project. This would probably be in April – date to be finalised with the members.

Next Steps

ICB outlined the next steps in the project plan:-

1. Once the draft reports of the last two workshops have been approved by all the participants, they will be published on the CRIMALDDI website.
2. The CRIMALDDI Team will integrate the recommendations into a coherent and aligned set under a series of appropriate themes. These will connect the common themes from the workshops where appropriate. The themes will probably be very similar to those already identified for the presentation at the ASTMH Evening Event (see above).
3. The CRIMALDDI Team will then prioritise the recommendations at the next Management Meeting in January 2011.
4. The final report will then be drafted, bearing in mind the strong views of the EAG that this must advocate for the outputs from the project.
5. The EAG will meet to review and (hopefully) endorse the final report in April. In order to be able to involve US-based members, it was suggested that this meeting should be held at a time where other meetings would make it easier for members to attend in the UK. All US-based members would be invited to comment on the draft report by email if they could not attend the meeting in person.
6. It is hoped that there will then be an opportunity after the final EAG meeting to take the report to Brussels to present the findings to members of the Commission and other interested groups based there.
7. ICB & SAW (budget permitting) would visit each of the main funding and opinion forming organisations to present the findings of the project in more detail and answer questions during 2011.
8. 2-3 papers to follow up on the introductory paper in Malaria Journal summarising the findings of the project to be written for publication in 2011.

The EAG expressed satisfaction with this plan. It was suggested that a presentation at the July 2011 Gordon Conference in Lucca might be of value. SAW to pursue this with Patrick Duffy (Conference Chair).

ICB/icb
16 November 2010.

Chairman's Confirmation

This is to confirm that this report represents a fair summary of the discussions of the CRIMALDDI Expert Advisory Group held on 02 November 2010.

Simon Croft (Professor)
London School of Hygiene & Tropical Medicine
