# Supplementary material for: CRIMALDDI: a prioritized research agenda to expedite the discovery of new anti-malarial drugs
Source: Malar J. 2013 Nov 5;12:395. doi: 10.1186/1475-2875-12-395 (PMC3830512; doi:10.1186/1475-2875-12-395)
Supplement: Additional file 4 — CRIMALDDI Workstream No. 4. Stage-specific Screening Methods. [file 1475-2875-12-395-S4.pdf]

Antimalarial Drug Discovery
CRIMALDDI
Coordination, Rationalisation, Integration

CRIMALDDI CONSORTIUM

(Co-ordination, Rationalisation, & Integration of Antimalarial Drug Discovery & Development Initiatives)

EXPERT ADVISORY GROUP

MEETING No. 4

London: 14 March 2011

Page intentionally left blank

Attendees:

Expert Advisory Group	
Prof Simon Croft	London School of Hygiene & Tropical Medicine (Chair)
Prof John Adams	University of South Florida
Dr Laurent Fraisse	sanofi aventis
Dr Federico Gomez de las Heras	*formerly* GlaxoSmithKline
Dr Saman Habib	Central Drug Research Institute, Lucknow
Prof Odile Mercereau-Puijalon	Institut Pasteur
Prof Geoffrey Targett	London School of Hygiene & Tropical Medicine
CRIMALDDI Consortium	
Prof Steve Ward (SAW)	Liverpool School of Tropical Medicine (Scientific Co-ordinator)
Prof Kelly Chibale	University of Cape Town
Prof Christian Doerig	INSERM
Prof Simon Efange	University of Buea
Prof Michael Lanzer	University of Heidelberg
Prof Donatella Taramelli	University of Milan
Prof Henri Vial	CNRS
Ian Boulton (ICB)	Consultant – Project Coordinator
Susan Jones (SJ)	Liverpool School of Tropical Medicine (EU Projects Manager)
Tracy Seddon	Liverpool School of Tropical Medicine

Meeting Objectives

The EAG (as it had at its previous meetings) noted that its role was not one normally undertaken by an expert group as it was not reviewing the science of specific projects. Its role in this case is to ensure that overall initiative remains in line with its objectives and that the workshops are delivering on the key dimensions of CRIMALDDI. It was agreed that the recommendations of the Consortium to be presented would be reviewed against the following headings:-

- Progress: Progress of workstream against CRIMALDDI objectives;
- Co-ordination: Co-ordination of the outputs of the workshops with other initiatives;
- Process: Ensuring that the outputs pay attention to the drug discovery process.

Review of EAG Terms of Reference

The EAG reviewed its terms of reference (ToRs) as agreed at the first meeting in Washington (Nov 2009). These are:-

1. Review proposals for priorities and action plans in drug discovery
2. Advise on priorities not identified but needed
3. Advise on research groups not identified by Consortium
4. Endorse the final priorities and action plan
5. Ensure no conflict with GMAP, and advise on resolution of any conflicts

Review of Recommendations by Key Theme

SAW presented the findings and recommendations of the Consortium to the EAG. This is embedded below. The first draft of the written report of the Consortium had been circulated to the EAG as a pre-read and was also discussed alongside the presentation.

EAG Meeting 03-11 - final.ppt

[Double click icon to open presentation]

Attacking Artemisinin Resistance

The EAG asked that the report made clear that there are two distinct situations where "resistance" may be seen – the increased parasite clearance times or decreased clearance rates seen in SE Asia, and laboratory developed resistance. The latter can be developed to study resistance mechanisms. The report needs to make clear whether the recommendation is looking for stable parasite lines that can be used to study the SE Asia effect. More emphasis needs to be made of screening compounds with positive hits in HTS screens against the resistant strains from SE Asia. There may be a Quick Win in finding a late-stage development compound that is effective against resistant strains found in SE Asia.

The report presents the implication of resistance against peroxides as effectively preventing the further development or deployment of all peroxides. Comparison was drawn with β-lactams where different generations of these antibiotics could be developed to overcome resistance seen in earlier generations. This may be true with the peroxides as well.

The recommendations are not to overcome the situation in Cambodia but to develop new drugs that are effective against *P falciparum* even those resistant to current artemisinin derivatives.

Creating & Sharing Community Resources

The EAG wanted it to be made clear in the recommendations that highly detailed standard protocols for testing and for validating targets need to be submitted to the database. The Community needs to own these protocols and the database is a place where peer review of them can be undertaken. This needs to be a proactive approach. The equivalent process used by NCCLS (now CLSI, Clinical Laboratory Standards Institute) was recommended as a good model.

Delivering Enabling Technologies

A potential Quick Win is to make *P falciparum* sporozoites more available.

The EAG asked that the report clarified better exactly what was being recommended. There seemed to be a degree of duplication and the recommendations needed to be rewritten to make them clearer.

The EAG also felt that the report needs to make clear that these enabling technologies will lead to new drugs that will not necessarily meet the SERCAP target profile.

Exploiting HTS Hits Quickly

The EAG felt that the recommendation to expand the level of Med Chem support is not just to resynthesise the positive hits from the HTS screening, but also to then explore the chemical space around the hits to enable good lead optimisation. This needs to be made clearer in the report.

Identifying Novel Targets

The EAG felt that reference to the better use of "Current Methods" needed to make clear that this meant the better application of Systems Biology applied in a rational way. The report should broaden its recommendations to a wider use of phenotypes to different target pathways than just to increase activity against resistant strains. There is an opportunity to show more finesse than simply killing resistant parasites. Phenotypic screening may be a valuable tool in identifying novel targets.

The focus on the first and last 12 hours in the asexual blood stage was welcomed, but this will be difficult as these are less well understood periods.

It was suggested that some effort needs to be put into looking for common pathways between *P falciparum* and *P vivax*. There ought to be commonalities between the genomes that could be exploited in finding novel targets.

General Consideration of Recommendations & Report

The EAG was disappointed that the draft report did not capture the richness and excitement that they had seen in the separate reports on the 5 workshops. Too many of the recommendations needed to be explained in the presentation and were not obvious in the text. Since most people would only read the report, it is vital that the report is revised to improve its impact. The EAG were concerned that the format of the report was driving the content – trying to condense each Key Theme on to two pages. This should be avoided. The workshop reports were first rate and clearly identified the research gaps that needed to be addressed.

The report also needed to be harder hitting and to try and be attractive to the European Union. The EAG were concerned about reports of the lack of interest in funding future research by the EU into malaria.

The EAG recommended that the time to impact of the different Key Themes should be highlighted. It is likely that someone will ask which Themes are of higher priority than others.

The view was expressed that the report was not clear enough on what should be the role of the pharmaceutical companies. As currently written, the industry members of the EAG felt that it was too focused on what academic groups should be doing and it was not clear if industry had a role at all.

All workshop attendees should be listed in the report.

The EAG insisted that reference to the development of ookinete and gametocyte motility assays and to the use of Toxoplasma as a possible screen should be removed from the "Nice to Have" list of recommendations. They very strongly did not support their inclusion.

Dissemination Plans

The EAG endorsed the plans to disseminate the recommendations of the project as described. In particular, they re-emphasised the need to re-engage with the EU and to try to maintain EU funding for malaria in Framework Eight.

Progress vs. Objectives

The EAG concluded that the Consortium had met its objectives. It re-iterated the need to make sure that the richness of the findings was properly reflected in the reports and papers that publicise the results.

Chairman's Confirmation

This is to confirm that this report represents a fair summary of the discussions of the CRIMALDDI Expert Advisory Group held on 14 March 2011.

Simon Croft (Professor)
London School of Hygiene & Tropical Medicine
