# Supplementary material for: CRIMALDDI: a prioritized research agenda to expedite the discovery of new anti-malarial drugs
Source: Malar J. 2013 Nov 5;12:395. doi: 10.1186/1475-2875-12-395 (PMC3830512; doi:10.1186/1475-2875-12-395)
Supplement: Additional file 5 — CRIMALDDI Workstream No. 5. Using Chemistry to Understand Biology. [file 1475-2875-12-395-S5.pdf]

# CRIMALDDI

## WORKSTREAM No. 1

## *P. falciparum & P. vivax*
## NOVEL TARGETS & CLASSES

## Report of a Workshop held at Liverpool School of Tropical Medicine

## 25-26 October 2010

This page is intentionally left blank

## Participants:

| | |
|---|---|
| Prof Christian Doerig (Co-chair) | EPFL-INSERM |
| Prof Kelly Chibale (Co-chair) | University of Cape Town |
| | |
| Prof Steve Ward | Liverpool School of Tropical Medicine |
| Ian Boulton (Facilitator) | TropMed Pharma Consulting |
| Susan Jones | Liverpool School of Tropical Medicine |
| | |
| Prof Pietro Alano | Istituto Superiore di Santa |
| Dr Giancarlo Biagini | Liverpool School of Tropical Medicine |
| Prof Hagai Ginsburg | Hebrew University of Jerusalem |
| Dr Dean Goodman | University of Melbourne |
| Dr Clemens Kocken | Biomedical Primate Research Centre, Netherlands |
| Dr Didier Leroy | Medicines for Malaria Venture |
| Dr Alexis Nzila | University of Cape Town |
| Prof David Roos | University of Pennsylvania |
| Prof Phil Rosenthal | University of California, San Francisco |
| Dr Ilaria Russo | University of Perugia |
| Prof Jonathon Vennestrom | University of Nebraska |
| Prof Andrew Wilks | Monash University |
| Prof Paul Wyatt | University of Dundee |

## Introduction:

Despite increasing efforts and support for antimalarial drug R&D, globally antimalarial drug discovery and development still remains largely uncoordinated and fragmented. The current window of opportunity for large scale funding of R&D into malaria is likely to narrow in the coming decade due to a contraction in available resources caused by the current economic difficulties and new priorities (*e.g.* climate change). It is therefore essential that stakeholders are given well articulated action plans and priorities to guide judgements on where their resources can be best targeted.

The CRIMALDDI[1] Consortium (a European Union funded initiative) has been set up to develop, through a structured and logical process, a focused set of detailed priorities and recommendations to address these problems. In this way it is intended to help to guide the priorities for European antimalarial research in the coming decade. It will also contribute to the wider global discovery agenda setting, and contribute to the availability of new drug candidates in the short- and medium-term. The Consortium has identified 5 priority workstreams on which to focus:-

| Workstream No. | Short Name | Workstream Question | Workstream Leaders |
|---|---|---|---|
| 1 | *Pf* & *Pv* novel targets & classes | How to identify and exploit novel targets at all stages of the lifecycle of *P falciparum* & *P vivax*. | Christian Doerig<br>Kelly Chibale |

[1] The Coordination, Rationalisation, and Integration of antiMALarial Drug Discovery & development Initiatives

| Workstream No. | Short Name | Workstream Question | Workstream Leaders |
|---|---|---|---|
| 2 | Managing the wealth of new HTS data | Given the large number of molecular structures that have given positive hits in the HTS screens and which are to be release by the pharmaceutical industry (>20,000), how to develop systems to:-<br>Make the information available to the community in an accessible way;<br>Filter the structures with robust methods to identify those structures which are druggable and more promising starts for lead optimisation;<br>Allow the community to know who is working on which structures so that duplication can be avoided and resources not wasted unnecessarily. | Steve Ward<br>Ian Bathurst |
| 3 | Artemisinin resistance | How to identify the mechanism(s) of artemisinin resistance in order to be able to design strategies to overcome or avoid it through novel combinations or novel molecular designs that counter the mechanism(s). | Steve Ward<br>Michael Lanzer |
| 4 | Stage-specific screening methods | How to develop a complete set of robust and replicable screening methods that can be used to screen novel compounds for efficacy against the various stages of the Plasmodium parasite lifecycle. | Donatella Taramelli<br>Henri Vial |
| 5 | Using chemistry to understand biology | How to use the results of the whole cell screening of compounds for antimalarial activity as a way of gaining insights into the underlying targets of different drug classes and then use this information to identify novel targets. | Steve Ward<br>Ian Bathurst |

This is a report on the discussions and conclusions from Workshop No. 1 "*P. falciparum* & *P. vivax* Novel Targets & Classes".

# *The Challenge:*

Prof Kelly Chibale & Christian Doerig outlined the challenge that was in front of the workshop and some of the key issues that needed to be addressed.

## Background:

There is a clear need for the identification and development of antimalarials belonging to novel chemical classes, with novel modes of action (MoA) and new targets.

### Need for new targets / Modes of Action:

Since the publication of the malaria genome, there have been numerous efforts aimed at identifying and validating novel drug targets. Despite these efforts, the reality reflected in the overwhelming majority of antimalarial drug discovery projects is that much of the work is still focused on a limited number of historic targets (the folate and haemoglobin degradation pathways are prime examples), and primarily on the blood stage of the parasite's lifecycle. There is presumably a wealth of untapped targets in the parasite proteome – or rather proteome<u>s</u> – of the various developmental stages that can be targeted. Similarly, lipids in the parasite membranes may be a useful target.

### Need and opportunities for discovery of new chemical classes:

1. ***<u>"Malaria boxes".</u>***

It is anticipated that by the end of 2010 more than 5 million chemical entities will have been screened for antimalarial activity, based on whole cell screens of *P. falciparum* malaria. The screens have included fully-synthetic libraries (from both commercial sources and Pharma proprietary libraries) and natural product libraries. Activity has been established based on the ability to stop the growth of asexual *P. falciparum* malaria parasites over a 24- 48hr exposure period *in vitro.* About 20K compounds are now available that are known to have selective parasiticidal (versus mammalian cell killing) activity, but whose molecular targets are unknown. Many of these chemicals are likely to act through novel pharmacophores on novel molecular targets. This represents a unique resource that must be tapped, but it has its shortcomings.

2. **<u>Natural products.</u>**

The historic success of natural products in antimalarial chemotherapy is well known, and the chemical diversity that is derived from natural products is tremendous. Current drugs that have their foundation in historic natural products include quinine, artemisinin, hydroxynaphthoquinones, doxycycline, clindamycin, and azithromycin. However there are no compounds in Lead Optimisation or preclinical /clinical development that have originated from a natural product in recent years. Many natural products have shown potent antiplasmodial effects but for a variety of reasons these have not been progressed via Hit-to-Lead and Lead Optimization. In addition, there have been recent developments such as the availability of the whole parasite genome and chemical biology that warrant a revisit of the natural product route of drug discovery *vis-à-vis* target identification. Natural products are better suited in target identification because they generally span a different chemical space than synthetic compounds. In addition, natural products can be bioavailable with properties outside conventional Lipinski space and they have evolved to interact with biological molecules.

### Key Challenges & Questions:

The challenges in this context are many, and include:-

- Diversifying the life cycle stages that are targets for chemotherapeutic intervention. At present, the vast majority of drugs target the metabolically active trophozoite/schizont stages of the *P. falciparum* erythrocytic cycle.
    - Can biology/bio-informatics propose targets that act at different stages (e.g. early asexual erythrocytic stages, sexual or liver stages)?

    - Can cellular screening assays be developed on such different stages? If so, should the malaria boxes be screened on these? Or should new cellular screens be implemented (e.g. gametocytogenesis, early ring to trophozoite development) perhaps using high content analysis?
- Identify malaria box compounds acting on novel targets. It is likely that compounds with novel MoA are hidden within the malaria boxes.
    - Is it worth engaging on screening the malaria boxes on selected putative novel molecular targets?
    - What other strategies could be developed to address this challenge?
- Include *P. vivax* in the drug discovery pipeline. Malaria boxes have been selected on *P. falciparum*, but *P. vivax* requires urgent attention.
    - In the absence of a *P. vivax* culture system, would it be worthwhile to implement biochemical screens of the malaria boxes on putative targets?
    - Can the *P. cynomolgi* system represent a usable alternative for *P. vivax*?
- Accelerate and integrate the screening of natural products
    - What are the best ways to screen natural products (target vs. phenotypic whole cell screenings) and in what form?
    - What unique criteria, that are different from synthetic compounds, should we develop for selecting natural products for hit to lead medicinal chemistry progression?
    - How can we best utilize natural products in target identification?
    - What are the challenges facing antimalarial drug discovery efforts based on natural products?
    - Can we coordinate efforts in this area?

## *Workshop Question:*

How to identify and exploit novel targets at all stages of the lifecycle of *P falciparum* & *P vivax*.

## *Participants' Initial Reaction:*

Most anti-parasitic drugs work through either unknown or poorly characterised MoAs. There was some doubt expressed about whether a purely target-based approach to drug discovery would ultimately be the best approach. In addition it was recognised that taking a purely natural product approach had difficulties as the chemistry starting point was often hard to progress towards Candidate Selection. Areas of activity thought to be important to addressing this challenge included:-

- A more rational application of Proteomics tools.
- Clearer Target Validation strategies.
- New / Improved Target screens.
- Phenotypic screens that generate greater insights/information into the underlying biology.
- A greater role for Pharmacology and pharmacologists.

The current focus of target-based drug discovery has been largely directed towards the folate and haemoglobin degradation pathways in the parasite and to a lesser extent the mitochondria. People have returned to them as they are well characterised, validated and reasonably well understood from a biochemical perspective. However it is accepted that the parasite has many more potential targets and now is the time for a concerted effort from the community to try to identify and exploit them. The standard pharmaceutical industry approach has been target-based and the tools to do this and their limitations are now well-established. It is recognised that for malaria (and many other biological systems) there is often poor correlation between target-related activity and phenotypic 'cidal activity. There are several ways that this can manifest itself. Often potent inhibitors of pure targets in *in vitro* biochemical assays fail to exhibit any effect in whole cell screens. This can be due to drug access issues, biochemical redundancy within a particular pathway or an inability to inhibit to the point that compromises parasite viability. A more challenging occurrence is the situation where whole cell screens demonstrate potency which is due to multiple end-mechanisms (as suggested for quinolines and peroxides). Multi-target drugs are considered excellent for generating drugs that delay resistance

development but they may not fall out of targeted drug screens where we look at each target in isolation. There is an acceptance that the validation of new targets can be challenging and should not get in the way of the priority which is the next generation of new and effective drugs against key parasite life-stages. The focus should remain on identifying chemotypes that exert the required biological effect/phenotype and then get into chemistry as early as possible to progress to Hit-to-Lead and Lead Optimisation. The underlying biology is often a "nice to have" but may need to be given a lower priority as it is usually not on the critical path to product delivery.

A major challenge to a purely chemistry/compound driven approach however is the lack of tools to prioritise the large numbers of positive hits that have been identified in HTS campaigns. It is impractical to take 20,000 compounds through chemistry without further screening and prioritisation. There are a number of ways that this could be tackled and some of these are defined in workshop 2. The need for inexpensive but predictive assays was highlighted and it was noted that this had already been looked into in Workshop 4. Screens must be properly validated before being widely used to maintain credibility of the screening process. Working with *P vivax* is still a major problem and the community continues to rely on rodent or monkey-malaria models. While these are of value, they are not necessarily validated as being directly predictive of the human malaria.

Standard screening methods may also be misleading if the compound works in the later stages of the parasite lifecycle and development is normal until then. $IC_{50}$ values will be artificially high in this situation. It was recognised that a similar cellular response can be an indication of a similar MoA despite the specific drug target being different. Phenotypic screens have introduced a new paradigm into parasite drug discovery which delivers rapid answers. Identifying the target however is long and difficult.

There was a lively discussion about the appropriate speed of action for new antimalarials and therefore which part of the parasite lifecycle would be an appropriate point to target. Normally the demand is for a rapid speed of action which tends to de-emphasise those with delayed effects (*e.g.* anti-apicoplast/anti-mitochondrion transcription, general inhibition of protein synthesis). It was accepted that this pharmacological characteristic may not be such a barrier to deployment if the application was for prophylaxis or control programmes rather than treatment of acute disease. Similarly, irreversible changes in the parasite make for effective drugs and this should be a focus for novel targets. The residence time of the drug-target complex should also be considered in drug/target selection. It was noted that nowadays antimalarial drug combinations are the accepted norm and so drugs that have slower speed of action could be of value combined with rapid acting drugs. The speed of action of the artemisinins is attributed to their effect throughout much of the asexual erythrocytic cycle and notably their potent activity against the ring stage parasite. It was suggested that one area that should be focused on was targets and biochemical process or chemotypes that can be demonstrated to exert an effect within the first 12 hours of the asexual erythrocytic stage.

The increasing number of databases on genes and metabolic pathways offer opportunities to identify possible new target based approaches to be explored. The cross-correlation of this information can be a very valuable filter. Various examples were shown (*e.g.* identifying genes that are active in gametocytes but not in blood stages). Orthology-based screening between Plasmodium species may also give valuable insights into promising new targets. Similarly the orthology between Plasmodium and Toxoplasma can indicate where Toxoplasma models may be of value. The high level of conservation between *P falciparum* and *P vivax* genomes indicates that novel targets for vivax could be established from proven falciparum targets.

As the effort against malaria moves from the Control Phase (as defined the GMAP[2]) to Elimination, new parts of the parasite lifecycle beyond the blood stages will need to be targeted. Liver stages, sporozoites, and gametocytes were highlighted as being potentially important for the elimination phase.

[2] Global Malaria Action Plan

It was felt that chemists and biologists may prioritise compounds from different viewpoints and there needs to be alignment of approaches.

Natural products are an "exquisite" source of new drugs as Nature has screened orders of magnitude more compounds than has been possible with high-throughput screening campaigns (HTS). Their chemical space is closer to actual drugs than combinatorial chemistry libraries. The shared ancestry of apicoplexans and algae suggest that new "plant-like" targets exist beyond the apicoplast. This may be of value in identifying leads for less metabolically active stages in the parasite lifecycle (*e.g.* gametocytes, hypnozoites). The problem of the regulatory pathway with natural product mixtures was noted. The drive for low cost drugs also was a problem for natural products as they were not usually small molecules. However better semi-synthetic approaches (as has is being used for artemisinin) and the falling incidence of malaria reducing the quantities of compounds needed may reduce this problem.

There was general agreement that HTS campaigns using agrochemical company libraries should be undertaken as soon as possible.

The issue of publication and intellectual property protection was briefly raised but not pursued in depth. It was agreed that better communication and co-ordination throughout the community should be an area of focus (also conclusions from Workshops 2 & 5). The need for a clear and data-driven decision-making process on the priorities to be given to different targets was also raised and generally agreed with.

# *Proposal Development:*

## General Recommendations:

1. It was agreed that choices of priorities for funding in this field need to be made transparently based on all available data. The participants wanted to make sure that priorities were not distorted by the personal preferences of a few influential opinion leaders.
2. If there is going to be any progress on taking a target-based approach to finding novel antimalarials, then a priority needs to be the establishment of reporter systems for every stage of the parasite lifecycle. Certain stages of the lifecycle are higher priority for drug discovery & development since they have the most promise of producing good drug candidates to meet the community's needs. These should have priority for developing reporter systems.
3. To be of any value in identifying novel drug candidates, assays need to be fast and cheap. It was noted that this had been a topic in Workshop 4.
4. Targets must be properly validated and functional assays should be easily reproducible in more than one centre. The gold standard of target validation is complementation. Validation is often problematic, but it is important that all arguments to support a particular target must be credible and efforts made to increase the evidence base as time goes on. Correlation between cellular and enzyme assays is essential for target based projects
5. Resources should be put behind integrating and maintaining the various databases on genes and metabolic pathways that currently exist. This is a valuable resource for the community and a properly integrated information system would be an effective use of the community's efforts. Resourcing would not be excessive (no more than US$ 2 million / year for a comprehensive system – based on similar initiatives). This mirrors the recommendations of Workshop 2.
6. The Structural Genomics Consortium is resourced to obtain at least 300 crystal structures in the field of malaria. The participants and wider community were encouraged to nominate suitable candidates to take advantage of this resource.

It was agreed that it was of value to frame the conclusions under the various components or phases of the GMAP – Control & Elimination – in order to look at the specific attributes needed for drugs to be used in each phase. For drugs, the impact of each phase is as follows:

**Control:** Making a substantial impact on malaria through the treatment of acute symptomatic infections. In general, this is the treatment of the asexual blood stages of infection, which cause symptoms and will be when the patient presents for treatment.
**Elimination:** Using drugs to reduce and ultimately stop transmission through the elimination of asymptomatic parasite reservoirs in the human body and/or interrupting the parasite lifecycle elsewhere than the acute blood stage.

For the purposes of this workshop, it was agreed to focus on uncomplicated malaria. This remains where the major burden of disease is. Treatment of severe malaria and other approaches may be the subject of separate workshops if CRIMALDDI progresses to a Phase 2 and new drugs for interventions like Intermittent Presumptive Treatment (IPT) will probably be identified from the pool of possible treatment candidates based on pharmacokinetics and other relevant properties.

## GMAP Control Phase:

In the Control Phase of GMAP, the focus is on the treatment of symptomatic patients. This means the treatment of asexual blood stages of the parasite lifecycle.

### Finding Novel Drug Classes:

It was agreed that the most promising approach here remains the whole cell screening of compound libraries, and then further prioritising the positive hits in secondary screens. This process and the need for improved secondary screens were dealt with in some detail in Workshop 4. A wider range of libraries should be investigated, especially the compound banks from agrochemical companies.

### Finding Novel Targets:

Firstly the characteristics of a good target to be used in drug discovery were identified:-

**“Must Have” Characteristics:**

- The effect should be ‘cidal in 48 hours through either inhibition of the target or interaction of the chemical compound with the target.
- The target should be validated either chemically or genetically, both *in vitro* and *in vivo* at a credible stage of the lifecycle of the parasite.
- The target should be druggable, *i.e.* should be amenable to treatment with small molecules at low concentrations.
- There needs to be a reliable functional assay of the target.

**Nice to Have:**

- Orthology of the target in humans.
- Cellular assay of the effect of compounds on the target.
- A crystal structure of the target.

There was an in-depth discussion about the most appropriate point in the erythrocytic stage to target. A key requirement in most treatment target product profiles (TPPs) is rapid kill. The benefits of the artemisinins in producing such an effect can be attributed to their activity throughout all the ring stage. Other proven antimalarials are only active in the trophozoite stage. Similarly interfering with the schizont stage and interrupting the rupture and release of merozoites is a potentially promising point to attack the parasite but not yet exploited. Ideally a drug would work across all these stages if a suitable biochemical process can be identified.

It was agreed that priority should be given to characterising and identifying potential drug targets in the first 12 hours of the ring stage and the last 12 hours of the blood schizont stage. This will require work on elucidating the biology of these parts of the lifecycle. The workshop identified the following more detailed stages as priorities for further biological understanding that might lead to novel targets of most value for drug discovery:-

*Ring Stage:* Merozoite invasion → Formation of the parasitophorous vacuole & attendant transport pathways → Metabolic pathways in the trophozoite (especially haemoglobin degradation) and host cell remodelling.
*Schizonts:* Merozoite ontogeny (assembly of daughter parasites) → Egress mechanisms

The workshop agreed that it was reasonable at this stage to consider *P falciparum* as a suitable model for blood stage *P vivax* infections. However some work on the read-across between the two species would be useful to properly validate this assumption.

## GMAP Elimination Phase:

The major challenge to identifying new drugs and targets for this phase is the paucity of suitable platforms to study the underlying biology and to assay chemical libraries. This mirrors the conclusions from Workshop 4.

### Liver Stages:

The highest priority is to be able to understand the biology of the liver stages, especially the dormant stages that act as a reservoir for *P vivax* and so make it such a problem in elimination campaigns. It is not even clear how to define a hypnozoite. Without such understanding, current screening techniques are not really practical for reasonable throughput levels, and modes of action will be poorly understood. A high priority is to develop simple markers to identify hypnozoites from active infected hepatocytes (preferably in human liver cells). This would be a key step to developing screens against the dormant stages.

Priority should be given to the following aspects of the liver biochemistry:-

- Reactivation of hypnozoites in an *in vitro* model to allow testing of compounds which block reactivation or kill the dormant cells.
- Causes of dormancy and the reasons for liver stage parasites to "choose" between the active and dormant state.

Some "quick wins" were identified:-

- Investigate the orthology between Plasmodium and Toxoplasma bradyzoites.
- Investigate the activity of primaquine and tafenoquine against bradyzoites, to see how far one could use Toxoplasma as a model for Plasmodium.

There are some insights or leads that may be starting points for research:-

- Known efficacy of 8-aminoquinolines (primaquine and tafenoquine) against hypnozoites.
- The use of bio-informatics to identify potentially important genes and metabolic pathways (*NB*: transcript abundance does not automatically correlate to biochemical importance).
- Non-haem targets in the blood stage.
- Effect of antibiotic pre-treatment of apicoplasts.

It was felt that some areas could be eliminated from this work as not being of value in elucidating the underlying biochemistry of the liver stages quickly and so developing techniques of use in drug discovery:-

- Replication machinery.
- Folate metabolism.
- Mitochondrial action?

## Other Stages

There is interest in looking at other stages in the lifecycle of the parasite to see if interfering with these stages will add any additional benefit to targeting the erythrocytic and hepatic stages. The majority of participants agreed that looking for drugs that specifically targeted these stages – sporozoites and gametocytes – would not be productive, but that adding activity against these stages to blood and/or liver stage activity could be of value. However the workshop ran into the problem that the community has not yet properly set out what it requires for drugs to be primarily used in the Elimination Phase of GMAP. A detailed TPP, setting out what effects need to be seen in the patient or asymptomatic carrier of parasites, needs to be developed. This will then allow the characteristics of the compounds to be found in drug discovery to be worked out and then the most appropriate stages of the parasite lifecycle targeted. Without this filter, it was recognised that it is very difficult to justify work on these stages from a drug discovery perspective and then to filter and prioritise the different areas of potential research.

Gametocytes are a potentially interesting point to interrupt transmission of the parasite. This could be either through additional activity from a compound that is acting elsewhere in the lifecycle, or as a targeted agent given as follow-up to acute treatment to mop up residual parasitaemia (somewhat analogous to add-on treatment of primaquine in vivax to mop up hypnozoites). The value of this "exit" drug concept could be established in the TPP mentioned above. It was noted that there will be some ethical and practical issues in developing a transmission-blocking only drug. A purely anti-gametocyte drug would not directly benefit the patient and so would impose a high safety requirement on the compound. On the other hand, a drug targeting gametocytes exclusively (but not affecting asexual stages) would be a valuable asset in preventing the escape of genotypes that confer resistance against anti-asexual drugs; thus one of the main benefits of transmission-blocking drugs would be the protection (and thus lifespan extension) of co-administered curative drugs. Therefore, the mechanisms of action of existing blood-stage active compounds against gametocytes should be investigated.

Possible targets to attack gametocytes were identified for possible further investigation:-

- Gametocytogenesis – specifically in *P. falciparum* this is a process with many metabolic processes active and so offering multiple points to disrupt the parasite.
- Egress mechanisms (especially proteases and lipid metabolism).
- The MoA of primaquine against gametocytes. Does it have common biochemical targets at all stages of the parasite lifecycle?

There is currently no evidence based conclusion over which gametocyte stage (I – V) should be a priority for in-depth research. It was agreed that targeting interesting biochemistry later in the mosquito stages of parasite development would pose considerable technical and pharmacological challenges and as such these should be de-prioritised.

Similarly finding drugs that have activity against sporozoites might be of value. Sporozoite-active compounds would act in many ways like a parasite repellent. The TPP should clarify how this type of activity will be of demonstrable value in the field. It will be useful to screen all the potential drug candidates from current screening hits against sporozoites to see if there is additional activity to the major activity against blood and/or liver stages.

Finally, there was a brief discussion on the possibility of targeting host cell (erythrocyte or hepatocyte) enzymes that are required for parasite survival. Since recent research indicates that such potential host cell targets include enzymes that are well-established targets in other pathologies (*e.g.* protein kinases in cancer), a piggy-back approach might be implemented that would considerably speed up discovery/development. However, there was a near-consensus that additional fundamental research must be performed to validate this approach.

## Natural Products:

As mentioned above, natural products can be an “exquisite” tool for identifying leads. Natural products are often complex molecules and, as such, may not be suitable for development as drugs due to potentially high costs of manufacture. Screening of these molecules in whole cell assays however is of potentially great value. They are often “non-Lipinski” type compounds and, as such, can lead to the identification of new druggable targets that would be rejected if the Lipinski Rules were slavishly followed. They will also be of value in identifying polypharmacological MoAs. The metabolic activity identified with natural products can be used to define empirical targets without initially establishing a protein target. The protein may be determined later in the development process, but doing this can be lengthy. It was agreed that the naturally occurring “libraries” of natural products should not be ignored both in seeking novel chemical classes and also as tools to identify novel targets. They may have a better chance to produce hits in drug screening because they have evolved to interact with biological targets.

## *Next Steps:*

1. Ian Boulton to draft report to be reviewed by Christian Doerig & Kelly Chibale. Then entire workshop will have an opportunity to comment before it is published on CRIMALDDI website.
2. Planned presentation as part of a CRIMALDDI Evening Event at ASTMH Conference 2010 on 04 November 2010.
3. Paper written by members of the CRIMALDDI Consortium outlining results of the workshop as part of a series of papers detailing results of the CRIMALDDI Consortium’s work to be submitted for publication at end of the project. Depending on the way in which the series of papers is finally agreed upon, Workshop participants will be invited to be included as co-authors if this makes sense. All Workshop participants will be acknowledged in the relevant paper.

Ian C Boulton
27 October 2010.
