# Supplementary material for: CRIMALDDI: a prioritized research agenda to expedite the discovery of new anti-malarial drugs
Source: Malar J. 2013 Nov 5;12:395. doi: 10.1186/1475-2875-12-395 (PMC3830512; doi:10.1186/1475-2875-12-395)
Supplement: Additional file 6 — CRIMALDDI Consortium: Expert Advisory Group Meeting No. 1. [file 1475-2875-12-395-S6.pdf]

CRIMALDDI

WORKSTREAM No. 2

MANAGING THE WEALTH OF NEW HTS DATA

Report of a Workshop held at Liverpool School of Tropical Medicine

17 March 2010

This page is intentionally left blank

Participants:

Prof Steve Ward (Co-chair)	Liverpool School of Tropical Medicine
Dr Ian Bathurst (Co-chair)	Medicines for Malaria Venture (MMV)
Ian Boulton (Facilitator)	TropMed Pharma Consulting
Susan Jones	Liverpool School of Tropical Medicine
Dr Neil Berry	University of Liverpool
Dr Fred Bost	Scynexis
Dr Thierry Diagana	Novartis Institute for Tropical Diseases
Dr Sean Ekins	Collaborative Drug Discovery
Dr Val Gillett	University of Sheffield
Prof Kip Guy	St Jude's Children's Research Hospital
Dr Dave Hornby	University of Sheffield
Dr John Overington	European Molecular Biology Laboratories, Cambridge
Dr Tanya Parkinson	Pfizer
Dr Raman Sharma	University of Liverpool
Prof Donatella Taramelli	University of Milan

Introduction:

Despite increasing efforts and support for antimalarial drug R&D, globally antimalarial drug discovery and development still remains largely uncoordinated and fragmented. The current window of opportunity for large scale funding of R&D into malaria is likely to narrow in the coming decade due to a contraction in available resources caused by the current economic difficulties and new priorities (*e.g.* climate change). It is therefore essential that stakeholders are given well articulated action plans and priorities to guide judgements on where their resources can be best targeted.

The CRIMALDDI[1] Consortium (a European Union funded initiative) has been set up to develop, through a structured and logical process, a focused set of detailed priorities and recommendations to address these problems. In this way it is intended to help to guide the priorities for European antimalarial research in the coming decade. It will also contribute to the wider global discovery agenda setting, and contribute to the availability of new drug candidates in the short- and medium-term. The Consortium has identified 5 priority workstreams on which to focus:-

Workstream No.	Short Name	Workstream Question	Workstream Leaders
1	*Pf* & *Pv* novel targets & classes	How to identify and exploit novel targets at all stages of the lifecycle of *P falciparum* & *P vivax*.	Christian Doerig<br>Kelly Chibale

[1] The Coordination, Rationalisation, and Integration of antiMALarial Drug Discovery & development Initiatives

Workstream No.	Short Name	Workstream Question	Workstream Leaders
2	Managing the wealth of new HTS data	Given the large number of molecular structures that have given positive hits in the HTS screens and which are to be release by the pharmaceutical industry (>20,000), how to develop systems to:- Make the information available to the community in an accessible way; Filter the structures with robust methods to identify those structures which are druggable and more promising starts for lead optimisation; Allow the community to know who is working on which structures so that duplication can be avoided and resources not wasted unnecessarily.	Steve Ward<br>Ian Bathurst
3	Artemisinin resistance	How to identify the mechanism(s) of artemisinin resistance in order to be able to design strategies to overcome or avoid it through novel combinations or novel molecular designs that counter the mechanism(s).	Steve Ward<br>Michael Lanzer
4	Stage-specific screening methods	How to develop a complete set of robust and replicable screening methods that can be used to screen novel compounds for efficacy against the various stages of the Plasmodium parasite lifecycle.	Donatella Taramelli<br>Henri Vial
5	Using chemistry to understand biology	How to use the results of the whole cell screening of compounds for antimalarial activity as a way of gaining insights into the underlying targets of different drug classes and then use this information to identify novel targets.	Steve Ward<br>Ian Bathurst

This is a report on the discussions and conclusions from Workshop No. 2 “Managing the Wealth of New HTS Data”.

The Challenge:

Dr Ian Bathurst outlined the challenge that was in front of the workshop and some of the key issues that needed to be addressed.

Background:

It is anticipated that by the end of 2010 more than 5 million chemical entities will have been screened for antimalarial activity, based on whole cell screens of *P. falciparum* malaria. The screens have included fully-synthetic libraries (from both commercial sources and Pharma proprietary libraries) and natural product libraries. Activity has been established based on the ability to stop the growth of asexual *P. falciparum* malaria parasites over a 24- 48hr exposure period *in vitro*. The outputs of these screens is likely to be placed in the public domain as indicated by the recent announcement from GSK[2], one of a number of companies, or institutions, who have either screened their libraries themselves or arranged with the Medicines for Malaria Venture (MMV) to have their libraries screened as part of an initiative driven and financed by MMV.

Based on the success rates to date there are likely to be more than 20,000 sub micromolar hits arising from these screens. The malaria genome contains about 5.5K genes of which less than 1% are likely to be druggable targets for chemotherapy. So we must conclude that many of the Hits identified will be targeting the same process. In the coming year or so we expect that the stage specificity of these Hits (i.e. activity against sexual stages and replicating and dormant liver stages) will be established as will their *in vitro* cellular therapeutic indices based on screens of representative mammalian cell lines.

The Challenge:

The process of taking a screening hit and progressing it through the lead identification, optimisation, and candidate selection process is expensive and time consuming. It is questionable whether a significant number of these screening Hits can be progressed through this pipeline with current resources without some significant prioritization. It is unclear at this time what level of detail will be put into the public domain or if anything other than chemical structure will be available to the community. Assay conditions differ between laboratories and so results are often difficult to replicate. While it may be possible for positive Hits to be put into the public domain, there are significant intellectual property and related issues in publishing negative results. It is estimated that as many as 66% of compounds screened may be proprietary. However these are needed in order to work out good structure-activity relationships (SARs). Are there ways of allowing research groups access to this proprietary information without putting it in the public domain but yet allowing robust work to be done on developing SAR relations and related algorithms? It is likely that there will be a significant level of replication of Hits between different compound libraries and de-replication of these is an issue. It is unlikely that any organization, including big Pharma, will be able to progress more than a handful of these hits into the development pipeline. It will be important to engage the entire academic community if we are rationally and efficiently to work our way through this wealth of data and turn these Hits into viable drugs for deployment.

The Workshop Goal:

The primary goal is to find ways to accelerate the rate at which these Hits become new drugs or are discarded based on good experimental evidence. By bringing together researchers with a diversity of expertise it is hoped to be able to generate strategies that could be used to help prioritize these Hits for further development. The malaria community needs to identify the information and tools that it will need to assess the value of the hits. It will need to identify transparent mechanisms that will allow groups to be aware of what templates are already under investigation by others and the templates that have been terminated because of a lack of developability. The expected development pathway (as required by MMV) needs to be formalized perhaps with guidelines about how to report data into a

[2] GSK opens door to malaria library. Chemistry World 2010 Mar; 7 (3): 21

central data-base and information of what the key data really is. It will need to think about the mechanisms that will allow appropriate communication between all interested parties, especially MMV.

Workshop Question:

Given the large number of molecular structures that have given positive hits in the HTS screens and which are to be release by the pharmaceutical industry (>20,000), how to develop systems to:-

- Make the information available to the community in an accessible way;
- Filter the structures with robust methods to identify those structures which are druggable and more promising starts for lead optimisation;
- Allow the community to know who is working on which structures so that duplication can be avoided and resources not wasted unnecessarily.

Participants' Reaction:

The participants were asked to give some initial reactions to the Challenge laid out in the introductory remarks. Some common themes emerged:-

- **Collaboration:** if anything is going to be done to improve the current rather disjointed sharing of information and duplication of work due to poor data sharing, then it will need to be achieved through some form of collaborative building of a shared database. A one-off data dump may have some short-term value but real value can only be achieved through an ongoing project that allows data-sharing.

- **Prioritisation:** there needs to be systems in place to filter the structures of positive Hits in order to remove those with poor drugability. This can be done with *in silico* filters if there is adequate understanding of the appropriate ADME, toxicological, and physiochemical properties needed for a good antimalarial. These may not be the same as for other infectious disease agents. To develop such screens however does require access to the negative Hits as well as the positive ones. There will need to be some general agreement on acceptable profiles for novel antimalarials to allow such filters to be built properly. While some properties are generally accepted as important (*e.g.* cost of goods, synthetic complexity, speed of parasite kill, stage-specific activity, solubility), the relative importance of these is not generally agreed upon. While MMV has developed robust target product profiles (TPPs) for novel antimalarials, these are looking at the profile from a clinical perspective. These requirements need to be developed into more detailed target candidate profiles (TCPs) that give guidance on the properties of the molecules needed to meet the TPP requirements. There also needs to be some agreement on which stages of the parasite's lifecycle should be targeted and should therefore be given priority. Also the community needs to be clear on where the priority for non-falciparum species should be placed.

- **Proprietary Information:** this is a considerable barrier to making all the necessary information available to the wider malaria community. Without patent protection, industrial organisations are very reluctant to release information on screening results (especially negative ones). It is impractical to patent every compound screened (even if it were allowed by the patenting authorities) but the information needs to be protected in case it has commercial value in other disease areas that have not yet been investigated. This is also a problem for the academic community, who also have the challenge of academic priority of publication to consider as well. It may be possible to release data to small groups under confidentiality managed through a custodian organisation (like MMV or TDR). Some participants were interested if a useful database could be built using properties of the compound ranked against a robust TCP without revealing the actual structure. A promising subset of potentially interesting compounds could then be given to one-or-more interested groups under confidentiality to work upon.

- **Data Storage Architecture:** the design of any database will be vital to its success. This is true both of how easy it will be to deposit data into the database, the security of the different types of data (proprietary *vs* non-proprietary), and the ease-of-use of the system for people accessing data. There are examples of well run databases that might be suitable models (*e.g.* ChemSpider). It is possible to structure databases to allow for three-or-more levels of access (*e.g.* confidential: collaboration: public access).

  Data curation is essential for the value of a database and this needs to be thought through from the start of the design. However the participants had mixed views about the degree of curation that should be allowed in case valuable information was lost. There is no ideal compound and so multiple filters may simply remove all compounds.

  The decision-making process needs to be well defined ahead of the design of the database in order to allow for the structure to reflect it and so simplify getting useful information from the database. The process could be illustrated as follows:-

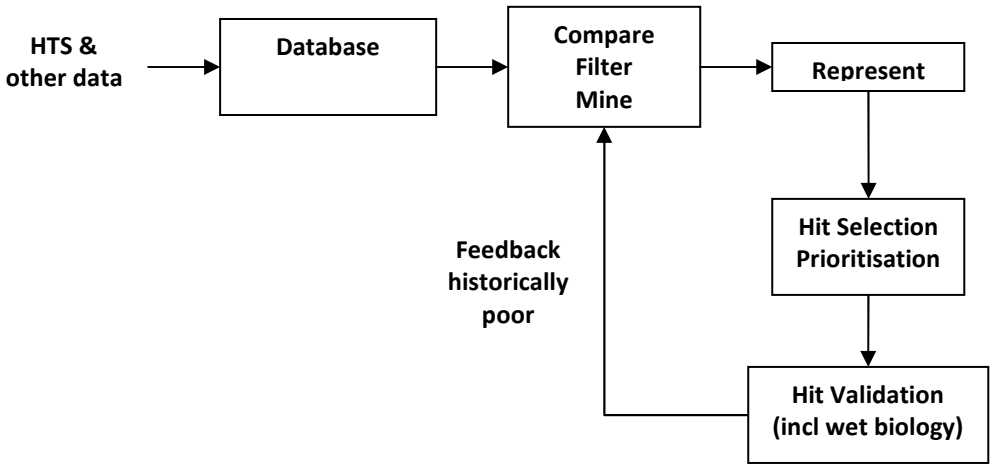

- **Involvement:** it is important to get a wide range of disciplines involved to integrate their findings with those of chemists and others in the databases. Experimental biologists were highlighted. It will be necessary to link the chemistry to biological pathways & regulators. Several participants noted the need to involve as wide a range of groups in accessing the HTS data, especially the small academic groups without existing strong connections to the pharma industry.

- **Data Utility:** there was concern about the inclusion of yeast-derived data from some members of the workshop, although others felt that this still have value and should be included. Targets identified from yeast-derived screens need to be validated in *P falciparum* before being widely used.

- **Novel Targets:** it is not necessary to identify the exact nature of a new target (if suspected) from a screening programme. The importance is to identify a new chemical space where whole cell screening has shown activity. The lack of good crystalline protein structures makes characterisation of the receptor and its bound substrate difficult. The lack of good structural data on novel targets has been the reason why the focus on specific pathways has been on those that are well characterised (*e.g.* dhfr, DODOH). This lack of good structural information underlines the continuing need for empirical wet biology to complement *in silico* analysis. It is also important to remember that one compound may have several targets and this can account for different results from different groups (poly-pharmacology).

- **Compound Availability:** it is not enough for the structures to be made available to research groups – there needs to be a mechanism to get access to the actual compounds. However

the synthesis of quantities of new compounds is not an unlimited resource and so there needs to be a mechanism to prioritise access to these powders.

Background Issues:

The participants identified several background issues that needed to be addressed as a proposal was developed to answer the question posed at the start of the workshop:-

- How to handle the problem of the intellectual property associated with the hits and misses found in screening programmes. Related to this was the challenge of academic authorship and ownership.
- How to reach out to the wider neglected tropical disease community to learn from and maybe integrate with successful initiatives.
- How to address the need for consortia to make any proposals from the workshop actually happen.
- How to keep all players involved and not build a system that eliminates the smaller groups (especially the academic groups).
- Who is going to do the work and how is it going to be funded.

Refining the Question:

The participants the refined the Question posed to them:

- How to make the information and the actual compounds available:
    - Who can have access
    - What can be made available, to whom, and when
    - Which screens & libraries can be included
    - What to do about negative results in screens
    - What is minimum data package needed for each compound
    - What format is the information available to be included
    - How to structure the database and how to structure the levels of access.
- What method to use to annotate the hits and compounds
    - Establish a standard set of assays aligned to the community's TPPs and TCPs derived from them
    - How to qualify the Hits and maintain this as an ongoing process
    - How will the Community resource and manage the database
    - How will the database be curated and data quality maintained
    - How will the database capture disease models and curate them to allow for the information to be assessed against these models
- How to enable the database to be used as a communication tool with the malaria community
    - Identify the number of groups working on each target
    - Establishing the feedback loop and maintaining its quality over time
    - Developing a mentoring process for groups to learn how to use the information available
    - Developing appropriate communication tools for each type of stakeholder
    - Aligned set of TPPs (showing clinical needs) with TCPs (showing compound profile needs)
    - Establishing workable rules over authorship and academic ownership to enable groups to deposit data in the database without risking losing priority over the results of their work.

Making the Information and Compounds Available:

The participants were in general agreement that a single database was needed as a common repository for all the HTS and related data to be made available to the malaria research community. It would need to be managed by a trusted and independent third party organisation (*e.g.* MMV) with strong internal governance that would be able to see all the data but could be trusted to maintain the necessary levels of confidentiality. This managing organisation could be responsible to a consortium of all stakeholders. It was foreseen as being an ongoing system that is constantly updated. Several participants noted that the current programme of making structures available through the process sponsored by MMV is only a one-off transfer of data. It would not necessarily be necessary to start from scratch in building a new database. Needed components could be added to one of the existing databases where HTS and related data is currently being deposited.

The workshop was concerned that GSK was making their information available on several websites. The risk is that each of the websites will curate the data differently and this will lead to confusion on the results that may come from use of this information. It would be preferable to have one central database as a stable reference repository from which others databases could either refer or draw information down from.

A recommended structure for the database will consist of three parts, each with different levels of access:-

Vault / Corporate	• Data lodged here will be embargoed from sharing with other organisations
Collaborative	• Data lodged here could be shared with other organisations once appropriate confidentiality and other agreements had been put in place
Open access	• Data lodged here would be freely available to anyone accessing the database. Everything that is not proprietary would be released through this part

The organisation managing the database, since it would see all the data (including the "Vault/Corporate" data) would be able to identify possible collaborations between groups. It would then be able to bring the groups together and try to arrange agreements whereby the data in question could be moved from "Vault/Corporate" to "Collaborative". However it was recognised that writing such collaborative agreements could be a challenge. Groups working on the same class of compounds could be brought together to pool results and encourage the early termination of work if the pooled results showed this to be appropriate. Complete transparency and open data sets usually goes beyond pharmaceutical companies' normal practice and will be a high hurdle to overcome. There will need to be education of the malaria community on this issue and the challenge that it represents. The open access part of the database will need a disclaimer about the availability (or not) of proprietary information.

The same managing organisation may not be appropriate for managing the process for identifying where actual compounds are available and identifying the priorities for access to this material. There needs to be a stable reference archive to ensure that the quality of the samples supplied to different groups is assured. A single resource person would need to vet requests to ensure that compounds were not wasted on unnecessary requests and that requesting organisations realised the scarcity of the materials. Access to this compound library needs to be transparent and the process and eligibility criteria can be agreed by the stakeholder consortium.

It is critical to the success of the proposed database that it is properly designed by specialists who have a proven track record in developing similar systems. The day-to-day running also needs to be put in the hands of a specialist team in this area. The managing organisation can have oversight of these specialists and manage the relationship(s).

The managing organisation needs to tackle the challenge of access to negative results from screens as their value to developing robust SARs could not be under-estimated.

The database will need to show the Standard Operating Procedures (SOPs) of each of the assays used by contributing organisations. There will need to be some mechanism for validating these assay methods to ensure only high quality data is lodged. In the longer term, the community will need to agree on a standard set of assays to be used to ensure data consistency.

Database as a Two-Way Communication Tool:

For the database to have any value, then it must be an interactive tool and not just a one-off repository for data. Communication must be embedded into the design. There are various tools (*e.g.* Wikis, SharePoint) to allow for interaction between groups. This will also allow the database to become a mentoring tool as some groups can help others with problems as they arise. However the academic participants were concerned that the design and operation of this communication tool must not compromise academic ownership of results and that credit for work done and results must be properly assigned and preserved. It was suggested that if data from the database is used in publications, then the originator must be added as an author to the paper. If this is not done then the infringer should be locked out of the system.

One concern about the design of the database as a communication tool was that it would be open to "mission creep". More and more functionality would be built into the design and it would then become either too difficult to use or would fail through becoming too complex. There was general agreement to keep it as simple as possible to meet a basic set of requirements, but that these need to be adequate for the database to be useful and so attract users. More functionality could be built in later as the need was identified and justified. There will need to be plenty of work done in the design phase by the database designers in identifying who wants to be involved with the database and what do they want to communicate about. This will then be built into the design from the start. Examples of good practice are PlasmoDB and DrugBank.

Read/write access needs to be properly controlled to avoid duplication of the same results.

Training on how to use the database properly will also need to be built into the design. This is also a motive for keeping the design simple to allow people with little time to learn the functionality to be able to use it and contribute data and opinions. It may make sense to have varying levels of interaction with the database depending on the interest and technical sophistication of different groups of users. There will also need to be some way of training the different specialities accessing the data to be able to use it properly (*e.g.* geneticists using structural biology data). Part of the training element of the database may well need to be on the role and challenges of IP in making data accessible and why some data is not open access.

The Consortium with oversight of the database will need to establish a task force to work with the designers to make the key decisions on the design. The database will need a feedback system to allow the community to comment on its working and design – a forum or similar part of the design. It was also suggested that there should be functionality to monitor access so that the database managers (*e.g.* MMV) can put groups with common interests in contact if they are not able to do this directly through the database.

Publicising the database will be key to its success. The participants recommended annual sessions on it – latest developments, upgrades, success stories – at annual meetings of the relevant societies (ACS, Medicinal Chemistry). The role of the Royal Society of Chemistry in running and publicising ChemSpider was highlighted. It might be possible to persuade journals to insist on depositing data

into the database as a prerequisite for publication of the results. Journal reviews were also recommended as communication vehicles both to encourage the lodging of data into the database and for people then to make use of it. Too many of these initiatives fail due to poor visibility in the community they are trying to serve. Hence a good communications plan to rollout the database and maintain awareness will be needed.

A key to success will have to be that there is adequate staffing for the database to be properly supported technically and in the communications area.

Data Curation:

There will need to be a clear procedure on how to annotate the data submitted. Empirical data needs to be submitted with a protocol describing how it was obtained and this should be peer reviewed by a suitably qualified person. In this way inadequate assay methods could be excluded and the data quality maintained. *In silico* predictions lodged in the database should be accompanied by the published algorithm used to derive them. One by-product of this would be to drive a process of standardising methodology and assays across the community.

A minimum dataset needs to be defined in order that rational decisions could be made using the data. The participants recommended that this would be:-

Minimum Requirement:

- IC_{50}
- CC_{50}

Preferred:

- Structure (if known and no proprietary issues)
- Solubility (kinetic)
- Permeability

Nice to Have:

- Compound purity

Calculated Data:

- Molecular weight
- Lipinski parameters

The data base should also have a “Community” space for each compound where *in silico* data can be added when available. It might be possible to set up the database to automatically calculate parameters like molecular weights (from the structure) and other “low hanging fruit”. All data on one structure needs to be co-localised. For non-active molecules, the IC_{50} could be shown as “inactive”. Initial information on enantiomers could be identified in a comments box. Links to other relevant databases (*e.g.* PubMed) can be built in.

It was agreed that the database should be for pure chemicals and not for natural product “broths”, which would need a separate structure for a database and would not form part of this exercise to inform small molecule drug discovery.

Through use of an accepted ontology for assay description, it should be possible to automatically control data quality at input and this will simplify data curation. Specified standards will be required. However this will not remove the need for a high quality curator working full-time on the project. There must be an agreed fixed naming convention to allow for easy access to information on one structure. De-replication of data needs to be done even-handedly to avoid criticism of favouritism by groups submitting data that is de-replicated.

Consortium Structure:

Clearly, a project like this will need to be ultimately run by a Consortium of relevant organisations with an interest in the area. The participants agreed on the following Consortium structure:-

- Leader — Essential to keep the stakeholders co-ordinated and drive the project forward. May well be the public face of the initiative.

- Stakeholders — They will own the project. Needs the following groups represented:-
  - Data generators (academia, industry, *etc.*)
  - MMV (as best organisation to manage the initiative on a day-to-day basis)
  - Funding agencies
  - Database managers and designers
  - Users (medicinal chemists, biologists, *etc.*)

This group would need to meet annually at least in order to maintain support for the project and make key decisions on the design and working of the database. These people will not be the same people as those actually running the database on a day-to-day basis.

The running of the Consortium will have to be transparent and decision-making seen to be reasonable.

Measures of Success:

How will the project know that is being successful? The measure of success that the participants agreed on was that this co-ordinated approach to data sharing was going to be more successful at generating new drug candidates than a simple one-off data dump into the public domain. The community needs to see that there is value in collaborating in the way proposed. However the Consortium, if it is set up, would have to put flesh on this concept and set more measurable parameters of success.

Next Steps:

1. Summary of this Workshop will be shared with participants in Workshop 5 ("Using Chemistry to Inform Biology")
2. Ian Boulton to draft report to be reviewed by Steve Ward & Ian Bathurst. Then entire workshop will have an opportunity to comment before it is published on CRIMALDDI website.
3. Planned presentation as part of a CRIMALDDI Symposium at a major conference – ASTMH 2010 is hoped for.
4. Paper written by Ian Boulton, Steve Ward, & Ian Bathurst outlining results of the workshop as part of a series of papers detailing results of the CRIMALDDI Consortium's work to be submitted for publication at end of the project. All Workshop participants who want to be included as co-authors will be added to the paper.
5. MMV to circulate details of their TPPs and TCPs to all participants.

Ian C Boulton
31 March 2010.
