# Supplementary material for: CRIMALDDI: a prioritized research agenda to expedite the discovery of new anti-malarial drugs
Source: Malar J. 2013 Nov 5;12:395. doi: 10.1186/1475-2875-12-395 (PMC3830512; doi:10.1186/1475-2875-12-395)
Supplement: Additional file 7 — CRIMALDDI Consortium: Expert Advisory Group Meeting No. 2. [file 1475-2875-12-395-S7.pdf]

CRIMALDDI

WORKSTREAM No. 3

ARTEMISININ RESISTANCE

Report of a Workshop held at the

Liverpool School of Tropical Medicine

05 October 2010

This page is intentionally left blank

Participants:

Prof Michael Lanzer (Co-Chair)	University of Heidelberg
Prof Steve Ward (Co-Chair)	Liverpool School of Tropical Medicine
Ian Boulton (Facilitator)	TropMed Pharma Consulting
Susan Jones	Liverpool School of Tropical Medicine
Prof Steffen Borrmann	KEMRI – Wellcome Trust Centre, Kilifi
Prof Ian Graham	CNAP, University of York
Dr Ian Hastings	Liverpool School of Tropical Medicine
Prof Gilbert Kokwaro	University of Nairobi
Prof Sanjeev Krishna	St George's Hospital, London
Prof Dennis Kyle	University of South Florida
Prof Odile Mercereau-Puijalon	Institut Pasteur
Prof Harald Noedl	Medical University, Vienna
Dr Alexis Nzila	University of Cape Town

Introduction:

Despite increasing efforts and support for antimalarial drug R&D, globally antimalarial drug discovery and development still remains largely uncoordinated and fragmented. The current window of opportunity for large scale funding of R&D into malaria is likely to narrow in the coming decade due to a contraction in available resources caused by the current economic difficulties and new priorities (*e.g.* climate change). It is therefore essential that stakeholders are given well articulated action plans and priorities to guide judgements on where their resources can be best targeted.

The CRIMALDDI[1] Consortium (a European Union funded initiative) has been set up to develop, through a structured and logical process, a focused set of detailed priorities and recommendations to address these problems. In this way it is intended to help to guide the priorities for European antimalarial research in the coming decade. It will also contribute to the wider global discovery agenda setting, and contribute to the availability of new drug candidates in the short- and medium-term.

[1] The Coordination, Rationalisation, and Integration of antiMALarial Drug Discovery & development Initiatives

The Consortium has identified 5 priority workstreams on which to focus:-

Workstream No.	Short Name	Workstream Question	Workstream Leaders
1	*Pf* & *Pv* novel targets & classes	How to identify and exploit novel targets at all stages of the lifecycle of *P falciparum* & *P vivax*.	Christian Doerig<br>Kelly Chibale
2	Managing the wealth of new HTS data	Given the large number of molecular structures that have given positive hits in the HTS screens and which are to be release by the pharmaceutical industry (>20,000), how to develop systems to:-<br>Make the information available to the community in an accessible way;<br>Filter the structures with robust methods to identify those structures which are druggable and more promising starts for lead optimisation;<br>Allow the community to know who is working on which structures so that duplication can be avoided and resources not wasted unnecessarily.	Steve Ward<br>Ian Bathurst
3	Artemisinin resistance	How to identify the mechanism(s) of artemisinin resistance in order to be able to design strategies to overcome or avoid it through novel combinations or novel molecular designs that counter the mechanism(s).	Steve Ward<br>Michael Lanzer
4	Stage-specific screening methods	How to develop a complete set of robust and replicable screening methods that can be used to screen novel compounds for efficacy against the various stages of the Plasmodium parasite lifecycle.	Donatella Taramelli<br>Henri Vial
5	Using chemistry to understand biology	How to use the results of the whole cell screening of compounds for antimalarial activity as a way of gaining insights into the underlying targets of different drug classes and then use this information to identify novel targets.	Steve Ward<br>Ian Bathurst

This is a report on the discussions and conclusions from Workshop No. 3 “Artemisinin Resistance”.

The Challenge:

Prof Steve Ward & Michael Lanzer outlined the challenge that was in front of the workshop and some of the key issues that needed to be addressed.

Is there artemisinin resistant malaria? A summary of our current understanding

Artemisinins are potent, rapidly acting antimalarial drugs that are widely used as first line treatment in malaria endemic areas. To preserve their efficacy, the World Health Organization has recommended that artemisinins be deployed as combination therapies (ACT) together with partner drugs. For example, artesunate is combined with amodiaquine and mefloquine, and artemether with lumefantrine. Despite this precautionary measure, artemisinin resistance has emerged in western Cambodia and the bordering region with Thailand (1, 4, 5, 12, 13, 23), where the artemisinin derivative artesunate has been used unregulated as monotherapy since the mid 1970s. It is claimed that artemisinin resistance is still contained and does not seem to have spread (13) although data are lacking. Reassuringly although the artesunate-mefloquine combination used in Cambodia does not kill the human malaria parasite *Plasmodium falciparum* as quickly as it did in the past, it still remains effective in the treatment of malaria (4). Should resistance to artemisinins and ACTs heighten and should it spread, malaria-related infections and casualties are expected to rise beyond today's 200 million clinical cases and 1 million deaths annually since there is no other drug class licensed, or in development, that can match artemisinins' efficacy and safety.

What do we know about possible mechanisms?

Loss of susceptibility to the artemisinins is not reflected in traditional IC_{50} values and genomic analysis of failing parasites has not been very informative to date. The molecular basis of reduced artemisinin responsiveness is unclear. Artemisinins are endoperoxides that are thought to form free radicals that may kill malaria parasites by alkylating essential biomolecules (3). Alternatively, artemisinins may act by specifically inhibiting the parasite's endoplasmic Ca^{2+} ATPase PfATP6 (6). A single point mutation in PfATP6 can abolish artemisinin sensitivity, as shown in a heterologous system, which has led to the model that artemisinin resistance is brought about by mutational changes in the artemisinin binding site of PfATP6 (20). However, in *P. falciparum*, polymorphisms within PfATP6 do not seem to correlate with altered artemisinin responsiveness, as suggested by molecular epidemiological studies (4, 8, 24), although one study found a single amino acid polymorphism, outside the putative artemisinin binding site, in field isolates with a reduced artemisinin responsiveness (10). Further, allelic exchange mutants expressing wild-type or mutant PfATP6 did not show clear differences in artemisinin responsiveness (21).

Equally unclear is the contribution of the parasite's multi-drug resistance transporter PfMDR1 to artemisinin resistance. PfMDR1 resides at the digestive vacuolar membrane of the parasite, where it transports solutes, including antimalarial drugs, into the digestive vacuole (16). Polymorphisms within, and overexpression of, PfMDR1 affect its transport properties and, in turn, drug responses (14, 17-19). Artemisinin interferes with PfMDR1 transport function (15), and some studies have correlated altered artemisinin responsiveness with mutational changes within, and increased copy number of, *pfmdr1* (14, 18, 19), whereas other studies did not find evidence for such a correlation (4, 8, 10).

A third candidate gene implicated in altered artemisinin responsiveness is an ubiquitin C-terminal hydrolase. Mutations in this enzyme are associated with artemisinin resistance in the mouse malaria parasite *Plasmodium chabaudi* (7). The significance of this finding for human malaria parasites is unclear (7, 8).

Other studies have proposed that increased tolerance to artemisinin in *P. falciparum* is mediated by a quiescence mechanism, bringing about growth arrest at the ring stage (22). This hypothesis takes into account the puzzling finding that parasites isolated from patients displaying delayed parasite clearance rates do not seem to have increased *in vitro* IC_{50}-values to artemisinin derivatives (4) using

standard *in vitro* sensitivity assays. Contrasting with this finding, field isolates collected in Cambodia, French Guiana, and Senegal displayed a wide range in *in vitro* IC_{50}-values against artemether and artesunate (10), suggesting that at least under some *in vitro* conditions a broad range of IC_{50}-values can be measured, a possible indicator of reduced artemisinin responsiveness in some parasites.

Several studies have attempted to generate artemisinin resistant *P. falciparum* strains *in vitro*, however with mixed results (2, 7, 9, 11). While strains with increased artemisinin IC_{50}-values could initially be selected for, they were often unstable. One study reported the selection of artelinic acid resistant parasites over a period of three years (2). These parasites could subsequently be adapted to survive high artemisinin concentrations (2). More recently a stable artemisinin line has been established from the progeny of the Dd2/3D7 genetic cross (Lanzer et al – not sure of reference) which shows no change in sensitivity to other peroxides.

The Challenge:

The current issues and uncertainties about artemisinins all arise from the lack of a measurable phenotype *in vitro* and the lack of any predictive biochemical or genetic markers. This means that all studies are based on clinical trials in humans and the measurement of parasite clearance rates. At present, key unanswered questions include:

- What do we mean by artemisinin resistance
- Has it spread beyond its origin in S.E. Asia
- What potential mechanisms could account for the reduced parasite clearance rates
- How are we going to approach the challenge of developing genotypic/phenotypic markers of altered sensitivity (what technologies have not been tried)
- How should we evaluate cross resistance between peroxides (semi-synthetic and synthetic)
- What does a sensible containment (elimination) strategy look like
- How can we stop its emergence in the future

The Workshop Goal:

By bringing together researchers with a diversity of expertise, we hope to be able to generate experimental strategies that could be used to investigate and establish markers for artemisinin resistance which can then be used to design and identify novel chemotypes which will counter the emergence of resistant parasites.

Workshop Question:

With the situation outlined in the Challenge section, the workshop was asked to address the following question:-

- “How to identify the mechanism(s) of artemisinin resistance in order to be able to design strategies to overcome or avoid it through novel combinations or novel molecular designs that counter the mechanism(s)?”

Boundaries of the Discussion:

The focus of the discussion was restricted to aspects of the challenge that directly relate to the search for new drugs that could replace the existing range of artemisinin derivatives if-and-when they become of limited use due to the development of resistance by the parasite. It was not considered part of the scope of the workshop to discuss methods to contain any resistance that is currently seen in the patient population in Cambodia.

Participants' Initial Response to the Challenge & Question

The characterisation of the nature of artemisinin resistance is still a work-in-progress. Although there is evidence from SE Asia that *P falciparum* is showing signs of tolerance to artemisinin based drugs, there is still no clear evidence confirming the existence of strains of the parasite that are fully/highly resistant to the drug. ACT treatment failures are quite rare even in Pailin Cambodia. The issue is the increase in parasite clearance times (PCTs). This increase could have several explanations:-

- Altered clearance of dead parasites.
- Delayed parasite death ("Pyknotic parasites").
- Dormancy of a sub-population of parasites.
- A shift in the stage sensitivity profile to these drugs (i.e. early rings become insensitive)

In cases of clinical failure, a suitable assay is needed to clearly show if the failure is due to drug resistance. An *ex-vivo* assay may be the best/only approach at this time.

The lack of markers of altered sensitivity to artemisinins was highlighted. The challenges were identified as:-

- Access to parasites that have demonstrated slow PCT's *in vivo*
- The need for stable cell lines of parasites with reduced susceptibilities.
- Improved *in vitro* assays.
- Better understanding of alterations to spleen clearance.
- Identification of alterations to the parasite population associated with reduced susceptibility.

The lack of clarity around the mechanism of action (MoA) of artemisinin and if this is a single or multiple mode has been another factor hampering our ability to understand what is happening when PCT's increase. It is equally unclear whether the mechanism of resistance is directly related to the MoA or not.

There was some discussion as to whether the rapid metabolism of artesunate, dihydroartemisinin (DHA), and other derivatives to artemisinin in humans was sufficient evidence to assert that any problem identified with artemisinin would automatically apply to the derivatives. IC_{50} values have increased with DHA but not with artemether. The key property of artemisinin derivatives is their speed of action and therefore it was possible that the drugs had been effective before they were fully metabolised to artemisinin. If this is the case, then cross-resistance should not be automatically assumed. Work undertaken in Heidelberg indicates that artemisinin resistance is genetically determined, but there is no evidence from this work of cross resistance to other derivatives. This is important as there are a wide range of endoperoxide molecules that can be identified in, for example, Artemisia spp., which may have utility as antimalarials. There also does not appear to be any difference in drug concentrations inside the parasite between resistant and sensitive strains.

There is data that shows differences in baseline IC_{50} values between SE Asia and Africa and this may confuse discussions about the emergence of tolerance / resistance in Africa if the absolute values alone are considered. We will need to establish if there is a genuine shift in IC_{50} and PCT's in a particular place after artemisinin based drug selection/exposure. We will then need to establish if the phenotype is stable or not?

Based on studies of crossing the two strains of *P falciparum* with differential drug sensitivity characteristics (HB3 & Dd2), it appears that the resulting parasite progeny display a range of IC_{50} values. Furthermore under drug pressure the resulting phenotype was dependent of the starting IC50. Using drug exposure to select for increased resistance, those parasites with the lowest IC50's took many months to display a significantly altered sensitivity profile and this phenotype was unstable. In contrast parasites with a high starting IC50 developed significantly increased levels of resistance quickly. Furthermore this phenotype was stable in the absence of drug pressure

Recent work at Kilifi, Kenya has also confirmed that increased PCTs are not correlated to increased *in vitro* IC_{50} as one might expect. This may be due to the nature of the *in vitro* assay and the relative lengths of exposure to drug. Also the *in vitro* ignores the impact of host-parasite interactions. There was felt to be a need for a more appropriate assay to address this issue. It is clear that there needs to be a concerted effort to phenotype the parasites that are showing tolerance / resistance as soon as possible.

Another possibility to be considered is that the increased tolerance is due to dormancy being induced in the parasites. *P falciparum* parasites in the ring stage can have dormancy induced by exposure to artemisinin and this dormancy is readily reversible. The recovery rates are different with different strains and for different peroxide drugs. The recovered strains do not have increased IC_{50} values unless there is repeated exposure over a long period of growth/dormancy cycles. This is an artemisinin-specific phenomenon. This raises the possibility of two mechanisms – dormancy and more conventional resistance mechanisms. Dormant parasites may still be cleared by the spleen possibly with altered rates.

The data to-date shows a distribution of IC_{50} around a mean value. This is usually not due to a mutation in a single gene but could indicate changes in a range of background genes. The important prediction is that modelling indicates that changes in IC_{50} to artemisinins will have a profound effect on the efficacy of any combination.

It was pointed out that the discussion is always framed around PCT and IC_{50} values. However maximum kill rates (V_{max}) is another parameter that is of relevance and it would be interesting to look at the variation in V_{max} among sensitive and resistant parasite strains.

Artemisinin resistance is not seen in Bangladesh despite use of monotherapy there. It was suggested that this is evidence that the use monotherapy in itself is not the cause of resistance, but it may be actually due to the widespread deployment of ACTs in Cambodia that is driving the reduced PCTs seen there.

Defining Artemisinin Resistance:

The workshop agreed that the definition of artemisinin resistance must start with a clinical one, based upon treatment failure and increased PCT. It is important not to incorrectly directly relate clinical resistance (measured through treatment failure) to parasite resistance (increased PCT and IC_{50}). There is a leap-of-faith in directly associating increased PCT with increased treatment failure. The next step must be to find suitable and practicable markers of reduced susceptibility. These tools need to be able to identify key parameters and they must be of practical use in drug screening programmes, preferably through simple *in vitro* assays.

Establishing Tools & Markers:

The current concerns about artemisinin resistance have shown that the classical tools used to identify resistance are inadequate. To date we have tried to characterise altered susceptibility using approaches that worked for quinolines and antifolates. We need to consider alternatives to the standard 48/72hour *in vitro* test. Parasite resistance may result from a number of direct or indirect mechanisms which may or may not link with MoA.

Current difficulties in obtaining resistant parasites and establishing stable lines were considered to be a major hurdle to progress. It was accepted that we need to address this limitation as a priority.

International concern has been triggered by the shift in PCT observed in Cambodia, but this is impossible to measure except through clinical trials and so is not suitable as a screening tool. Patients are also not homogenous and this may complicate analysis of clinical response data. Treatment failure has always been associated with increased PCT. However increased PCT is not very specific marker of treatment failure and is influenced by non- parasite factors such as host immunity. The baseline PCT

for a particular geographical area also needs to be established before any meaningful measurement of PCT increase can be made.

Priorities for the community are:

- To define what change in PCT is considered biologically and therapeutically relevant as an *in vivo* phenotype
- Collect more representative data on the range of PCTs within populations and geographical areas in the absence of artemisinin resistance in order to establish a robust baseline from which to identify the emergence of problems
- Better define the pharmacodynamics of parasite kill. In particular the relationships between IC_{50}, IC_{99}, the slope of the Hill plot and the maximum kill rate (equivalent to a Vmax in enzyme kinetics) need to be established for parasites with differing susceptibilities.
- Precisely define the window of parasite killing across the 48hr erythrocytic cycle and develop novel methods for evaluation stage specific killing and or dormancy. In particular we need to develop assays that can assess drug activity against early ring parasites.
- Using appropriate parasites (those already collected or being collected from Cambodia) attempt to correlate PCT with the experimental *in vitro* parameters as described above.
- Define the molecular and cellular basis of artemisinin induced dormancy
- Systematically re-evaluate all of the *in vitro* assays available (and all variations thereof) in order to look for discriminatory phenotypes.
- In preparation for failure, to evaluate the speed of action and stage specificity of current HTS hits (currently 15.000-20,000 novel chemotypes) to identify new chemotypes with similar PD to the artemisinins. This should include evaluation against parasites with stable resistance to artemisinins (and other antimalarials) and parasites arising from resistance "hot spots"
- Improve communication between active research groups in this area including sharing or materials and resources and adoption of standard procedures for the *in vivo* and *in vitro* investigation of this phenomenon.
- In the absence of clearly identified resistant phenotypes there is a need for looking at a range of "omic" approaches to search for discriminatory tools and markers. Transcriptional work is underway but the results are not yet available. The value of proteomic and metabolomic approaches should be considered. The workshop agreed that two approaches should be taken to screening for resistance markers and these approaches needed to be undertaken in parallel given the urgency of finding out exactly what is happening. The approaches are:-

    1. *Hypothesis generation* through whole genome scans of resistant and sensitive parasites to generate testable hypotheses bout resistance mechanisms and markers of resistance.
    2. *Hypothesis testing* through identifying correlations between possible markers of resistance and suggested mechanisms.

Designing New Drugs to Counter Artemisinin Resistance:

Characteristics of Single Agent and Combinations

The key characteristic of artemisinin is the early and rapid killing of parasites. The rapid kill is associated with rapid clinical effects. This is due to the effect throughout the ring stages (0-6, 6-12 h post invasion). Early ring stage activity will need to be retained in any replacement chemotypes, but without losing the broad stage MoA of current artemisinins.

Currently artemisinins are characterised by a short $T_{½}$. Ideally an alternative to current artemisinins should have a $T_{½}$ more closely aligned with those of its partner drugs. Other ideal characteristics were recommended to be:-

- No interaction with pfmdr and pfcrt enzymes.
- Lack of induction of dormancy in the parasites.

- No recrudescence found in a single agent situation.
- MoA should be independent of that of current artemisinins.

New drug compounds should retain activity against all clinically relevant resistant strains of *P falciparum*, and it would be nice also to have activity outside the blood stage.

Novel combinations should have the following characteristics:-

- All components should retain full efficacy against all clinically relevant strains of *P falciparum*. In the past, combinations have been potentially compromised because one component has already been used as monotherapy in clinical practice.
- Matched PKs is ideal but not essential. The key requirements are parasite kill and cure rates.
- Complementary PD is essential. The combination should kill across the entire parasite lifecycle. MoAs should ideally be synergistic.
- The combination should have coverage of at least one week from a 3-day or less course of treatment. This will then give coverage over at least 3 complete cycles of parasite replication.

Chemical Strategies:

Defining chemical strategies to address the challenge of finding alternatives to artemisinin derivatives is complicated by the lack of understanding of the mechanisms at play. There is urgency to develop stable laboratory models of resistance and identification of resistance phenotypes. These would then help to develop structure-activity relationships that could inform chemical strategies.

Co-Ordination with Related Initiatives:

Several organisations and initiatives were identified as being of important for CRIMALDDI to stay in contact with in order to avoid fragmentation of efforts in this area:-

NIH (Chris Plowe & Nick Fairhurst)
WWARN (Carol Sibley, Francois Benoit-Vical)
WHO-GMP (Pascal Ringwald)
Sanger Institute (Dominic Kwiatkowski)
Medicines for Malaria Venture
Wellcome Trust
Medical Research Council
Bill & Melinda Gates Foundation

Next Steps:

1. Ian Boulton to draft report to be reviewed by Steve Ward & Michael Lanzer. Then entire workshop will have an opportunity to comment before it is published on CRIMALDDI website.
2. Planned presentation as part of the CRIMALDDI Evening Event on 04 November at the ASTMH Annual Conference.
3. Paper written by members of the CRIMALDDI Consortium outlining results of this workshop as part of a series of papers detailing overall results of the CRIMALDDI Consortium's work to be submitted for publication at end of the project. All Workshop participants who want to be included as co-authors will be added to the paper.

Ian C Boulton
09 October 2010.

References:

1. **Alker, A. P., P. Lim, R. Sem, N. K. Shah, P. Yi, D. M. Bouth, R. Tsuyuoka, J. D. Maguire, T. Fandeur, F. Ariey, C. Wongsrichanalai, and S. R. Meshnick.** 2007. Pfmdr1 and in vivo resistance to artesunate-mefloquine in falciparum malaria on the Cambodian-Thai border. Am J Trop Med Hyg **76:**641-7.
2. **Chavchich, M., L. Gerena, J. Peters, N. Chen, Q. Cheng, and D. E. Kyle.** 2010. Role of pfmdr1 amplification and expression in induction of resistance to artemisinin derivatives in Plasmodium falciparum. Antimicrob Agents Chemother **54:**2455-64.
3. **Cui, L., and X. Z. Su.** 2009. Discovery, mechanisms of action and combination therapy of artemisinin. Expert Rev Anti Infect Ther **7:**999-1013.
4. **Dondorp, A. M., F. Nosten, P. Yi, D. Das, A. P. Phyo, J. Tarning, K. M. Lwin, F. Ariey, W. Hanpithakpong, S. J. Lee, P. Ringwald, K. Silamut, M. Imwong, K. Chotivanich, P. Lim, T. Herdman, S. S. An, S. Yeung, P. Singhasivanon, N. P. Day, N. Lindegardh, D. Socheat, and N. J. White.** 2009. Artemisinin resistance in Plasmodium falciparum malaria. N Engl J Med **361:**455-67.
5. **Dondorp, A. M., S. Yeung, L. White, C. Nguon, N. P. Day, D. Socheat, and L. von Seidlein.** 2010. Artemisinin resistance: current status and scenarios for containment. Nat Rev Microbiol **8:**272-80.
6. **Eckstein-Ludwig, U., R. J. Webb, I. D. Van Goethem, J. M. East, A. G. Lee, M. Kimura, P. M. O'Neill, P. G. Bray, S. A. Ward, and S. Krishna.** 2003. Artemisinins target the SERCA of Plasmodium falciparum. Nature **424:**957-61.
7. **Hunt, P., A. Afonso, A. Creasey, R. Culleton, A. B. Sidhu, J. Logan, S. G. Valderramos, I. McNae, S. Cheesman, V. do Rosario, R. Carter, D. A. Fidock, and P. Cravo.** 2007. Gene encoding a deubiquitinating enzyme is mutated in artesunate- and chloroquine-resistant rodent malaria parasites. Mol Microbiol **65:**27-40.
8. **Imwong, M., A. M. Dondorp, F. Nosten, P. Yi, M. Mungthin, S. Hanchana, D. Das, A. P. Phyo, K. M. Lwin, S. Pukrittayakamee, S. J. Lee, S. Saisung, K. Koecharoen, C. Nguon, N. P. Day, D. Socheat, and N. J. White.** 2010. Exploring the contribution of candidate genes to artemisinin resistance in Plasmodium falciparum. Antimicrob Agents Chemother **54:**2886-2892.
9. **Inselburg, J.** 1985. Induction and isolation of artemisinin-resistant mutants of Plasmodium falciparum. Am J Trop Med Hyg **34:**417-8.
10. **Jambou, R., E. Legrand, M. Niang, N. Khim, P. Lim, B. Volney, M. T. Ekala, C. Bouchier, P. Esterre, T. Fandeur, and O. Mercereau-Puijalon.** 2005. Resistance of Plasmodium falciparum field isolates to in-vitro artemether and point mutations of the SERCA-type PfATPase6. Lancet **366:**1960-3.
11. **Jiang, G. F.** 1992. [In vitro development of sodium artesunate resistance in Plasmodium falciparum]. Zhongguo Ji Sheng Chong Xue Yu Ji Sheng Chong Bing Za Zhi **10:**37-9.
12. **Noedl, H., Y. Se, K. Schaecher, B. L. Smith, D. Socheat, and M. M. Fukuda.** 2008. Evidence of artemisinin-resistant malaria in western Cambodia. N Engl J Med **359:**2619-20.
13. **Noedl, H., D. Socheat, and W. Satimai.** 2009. Artemisinin-resistant malaria in Asia. N Engl J Med **361:**540-1.
14. **Reed, M. B., K. J. Saliba, S. R. Caruana, K. Kirk, and A. F. Cowman.** 2000. Pgh1 modulates sensitivity and resistance to multiple antimalarials in Plasmodium falciparum. Nature **403:**906-9.
15. **Rohrbach, P., C. P. Sanchez, K. Hayton, O. Friedrich, J. Patel, A. B. Sidhu, M. T. Ferdig, D. A. Fidock, and M. Lanzer.** 2006. Genetic linkage of pfmdr1 with food vacuolar solute import in Plasmodium falciparum. Embo J **25:**3000-11.
16. **Sanchez, C. P., A. Dave, W. D. Stein, and M. Lanzer.** 2010. Transporters as mediators of drug resistance in Plasmodium falciparum. Int J Parasitol.
17. **Sanchez, C. P., A. Rotmann, W. D. Stein, and M. Lanzer.** 2008. Polymorphisms within PfMDR1 alter the substrate specificity for anti-malarial drugs in Plasmodium falciparum. Mol Microbiol **70:**786-98.
18. **Sidhu, A. B., A. C. Uhlemann, S. G. Valderramos, J. C. Valderramos, S. Krishna, and D. A. Fidock.** 2006. Decreasing pfmdr1 copy number in plasmodium falciparum malaria heightens susceptibility to mefloquine, lumefantrine, halofantrine, quinine, and artemisinin. J Infect Dis **194:**528-35.

19. **Sidhu, A. B., S. G. Valderramos, and D. A. Fidock.** 2005. pfmdr1 Mutations contribute to quinine resistance and enhance mefloquine and artemisinin sensitivity in Plasmodium falciparum. Mol Microbiol **57:**913-26.
20. **Uhlemann, A. C., A. Cameron, U. Eckstein-Ludwig, J. Fischbarg, P. Iserovich, F. A. Zuniga, M. East, A. Lee, L. Brady, R. K. Haynes, and S. Krishna.** 2005. A single amino acid residue can determine the sensitivity of SERCAs to artemisinins. Nat Struct Mol Biol **12:**628-9.
21. **Valderramos, S. G., D. Scanfeld, A. C. Uhlemann, D. A. Fidock, and S. Krishna.** 2010. Investigations into the Role of the Plasmodium falciparum SERCA (PfATP6) L263E Mutation in Artemisinin Action and Resistance. Antimicrob Agents Chemother.
22. **Witkowski, B., J. Lelievre, M. J. Barragan, V. Laurent, X. Z. Su, A. Berry, and F. Benoit-Vical.** 2010. Increased tolerance to artemisinin in Plasmodium falciparum is mediated by a quiescence mechanism. Antimicrob Agents Chemother **54:**1872-7.
23. **Wongsrichanalai, C., and S. R. Meshnick.** 2008. Declining artesunate-mefloquine efficacy against falciparum malaria on the Cambodia-Thailand border. Emerg Infect Dis **14:**716-9.
24. **Zhang, G., Y. Guan, B. Zheng, S. Wu, and L. Tang.** 2008. No PfATPase6 S769N mutation found in Plasmodium falciparum isolates from China. Malar J **7:**122.
