# Supplementary material for: CRIMALDDI: a prioritized research agenda to expedite the discovery of new anti-malarial drugs
Source: Malar J. 2013 Nov 5;12:395. doi: 10.1186/1475-2875-12-395 (PMC3830512; doi:10.1186/1475-2875-12-395)
Supplement: Additional file 8 — CRIMALDDI Consortium: Expert Advisory Group Meeting No. 3. [file 1475-2875-12-395-S8.pdf]

CRIMALDDI

WORKSTREAM No. 4

STAGE-SPECIFIC SCREENING METHODS

Report of a Workshop held at the World Health Organisation

26 May 2010

This page is intentionally left blank

Participants:

Prof Donatella Taramelli (Co-chair)	Università di Milano
Prof Henri Vial (Co-chair)	Centre National de la Recherche Scientifique, University of Montpellier
Ian Boulton (Facilitator)	TropMed Pharma Consulting
Tracy Seddon	Liverpool School of Tropical Medicine
Dr Jose Garcia-Bustos	GlaxoSmithKline
Dr Foluke Fakorede	TDR
Prof Annette Habluetzel	Università di Camerino
Dr Marcel Kaiser	Swiss Tropical and Public Health Institute
Dr Sumalee Kamchonwongpaisan	National Centre for Genetic Engineering & Biotechnology, Bangkok
Prof Clemens Kocken	Biomedical Primate Research Centre, Netherlands
Dr Didier Leroy	Medicines for Malaria Venture
Prof Louis Maes	University of Antwerp
Prof Maria Manuel Mota	Universidade de Lisboa
Prof Maurizio Muraca	Osepadale Pediatrico Bambino Gesu
Dr Solomon Nwaka	TDR
Dr Bernadette Ramirez	TDR
Prof Robert Sinden	Imperial College, London
Dr Georges Snounou	Université Pierre et Marie Curie/INSERM UMR S 945, Paris
Prof Roberta Spaccapelo	Università di Perugia
Dr Chairat Uthaipibull	National Centre for Genetic Engineering & Biotechnology, Bangkok
Dr Sergio Wittlin	Swiss Tropical and Public Health Institute

Introduction:

Despite increasing efforts and support for antimalarial drug R&D, globally antimalarial drug discovery and development still remains largely uncoordinated and fragmented. The current window of opportunity for large scale funding of R&D into malaria is likely to narrow in the coming decade due to a contraction in available resources caused by the current economic difficulties and new priorities (*e.g.* climate change). It is therefore essential that stakeholders are given well articulated action plans and priorities to guide judgements on where their resources can be best targeted.

The CRIMALDDI[1] Consortium (a European Union funded initiative) has been set up to develop, through a structured and logical process, a focused set of detailed priorities and recommendations to address these problems. In this way it is intended to help to guide the priorities for European antimalarial research in the coming decade. It will also contribute to the wider global discovery agenda setting, and contribute to the availability of new drug candidates in the short- and medium-term.

[1] The Coordination, Rationalisation, and Integration of antiMALarial Drug Discovery & development Initiatives

The Consortium has identified 5 priority workstreams on which to focus:-

Workstream No.	Short Name	Workstream Question	Workstream Leaders
1	*Pf* & *Pv* novel targets & classes	How to identify and exploit novel targets at all stages of the lifecycle of *P falciparum* & *P vivax*.	Christian Doerig<br>Kelly Chibale
2	Managing the wealth of new HTS data	Given the large number of molecular structures that have given positive hits in the HTS screens and which are to be release by the pharmaceutical industry (>20,000), how to develop systems to:-<br>Make the information available to the community in an accessible way;<br>Filter the structures with robust methods to identify those structures which are druggable and more promising starts for lead optimisation;<br>Allow the community to know who is working on which structures so that duplication can be avoided and resources not wasted unnecessarily.	Steve Ward<br>Ian Bathurst
3	Artemisinin resistance	How to identify the mechanism(s) of artemisinin resistance in order to be able to design strategies to overcome or avoid it through novel combinations or novel molecular designs that counter the mechanism(s).	Steve Ward<br>Michael Lanzer
4	Stage-specific screening methods	How to develop a complete set of robust and replicable screening methods that can be used to screen novel compounds for efficacy against the various stages of the Plasmodium parasite lifecycle.	Donatella Taramelli<br>Henri Vial
5	Using chemistry to understand biology	How to use the results of the whole cell screening of compounds for antimalarial activity as a way of gaining insights into the underlying targets of different drug classes and then use this information to identify novel targets.	Steve Ward<br>Ian Bathurst

This is a report on the discussions and conclusions from Workshop No. 4 "Stage-specific screening methods".

The Challenge:

Prof Henri Vial outlined the challenge that was in front of the workshop and some of the key issues that needed to be addressed.

Background:

In the context of the malaria control and elimination programs, drugs will continue to be used to treat acute malaria and prevent complications in vulnerable groups. However, at present most of the research for new drug continues to be focused on treating the blood stage of the parasite's life cycle. This is largely due to the fact that this was the only stage where we have had robust and reproducible screening methods to discover both activity and mode of action of new compounds. New strategies for malaria elimination imply a very strong commitment to prevent both the disease but also the infection caused by Plasmodium spp., to eliminate dormant malaria parasites from the liver and to prevent malaria transmission in addition to blood stage treatment. Thus, new drugs should be able to cure asymptomatic infections, relapsing liver stages and prevent transmission. This means that antimalarial drugs will be essential tools at all stages of malaria elimination toward eradication, including both the early "attack" phase to drive down transmission and later stages of maintaining interruption of malaria transmission, preventing reintroduction of malaria, and eliminating the last residual foci, *I.e*. radical cure of all life cycle stages of all five human malaria species.

Malaria eradication is therefore even more demanding since it will require drugs that can accomplish complete "eradication" of all malaria parasites from the bodies of infected humans. This includes the very low levels of parasites that cause no symptoms but which may still be a source of transmission. For *P. falciparum*, this entails the elimination of all persistent asexual or sexual blood stage forms, and, most importantly, the long-lived stage five *P. falciparum* gametocyte. For *P. vivax* malaria, radical cure includes elimination of all persistent asexual blood stage forms, but, most importantly, the long-lived hypnozoites in the liver. Safe and effective drugs that eliminate the mature stage IV *P. falciparum* gametocytes and the hypnozoites of *P. vivax* will dramatically improve prospects for eradication. Presently, 8-aminoquinolines such as primaquine are the only known drug class to be effective against both *P. vivax* hypnozoites and *P. falciparum* stage five gametocytes. However we have been hindered in our search for these drugs by the absence of robust, reproducible, and easy to use screening methods to identify activity in potential new compounds and classes.

Finally prophylaxis reflects the need to prevent new infection of naive subjects or re-infection of individuals already cured. Unfortunately we again have not had methods for screening novel compounds to identify promising preventative activity.

It is anticipated that million chemical entities could be screened for antimalarial activity. The screens may include fully synthetic libraries (from both commercial sources and Pharma proprietary libraries) and natural product libraries.

The Challenge:

In the context of eradication, new classes of antimalarial drugs active against all stages of all species of human malaria (either individually or through combinations with other drugs) are essential components for the progressive elimination of malaria from endemic countries. Ideally, they should affect the five human malaria species**.** The complexity of the life cycle is both a challenge and an opportunity as it gives us multiple potential targets to attack, if only we can identify them. Research is needed to identify these additional vulnerabilities in the parasites' biology.

These new drugs will not be identified without new tools, developed specifically for testing vulnerabilities of all the stages of the parasite life cycle to new type of effectors or drugs, and large scale assays for screening of potential drugs chemicals against new targets. These assays are essential to ultimately achieve this ambitious but eminently worthy goal of developing new drugs. Currently, this need remains unfilled and a top priority in the drug discovery process. This need is particularly

acute in light of the high number of compounds in the current drug development pipeline and the very limited number of existing tests for anti-relapse and transmission-blocking drugs.

The Workshop Goal:

By bringing together researchers with a diversity of expertise, we hope to be able to generate strategies to establish assays and models for screening compounds active against all the stages of the malaria parasites including liver and sexual stage parasites. If needed, we will identify and prioritize a set of knowledge gaps (including methodology gaps) and research questions that need to be addressed in order to test the many compounds that are needed to eliminate and ultimately eradicate malaria.

Workshop Question:

With the situation outlined in the Challenge section, the workshop was asked to address the following question:-

- "How to develop a complete prioritised set of robust and replicable screening methods that can be used to screen novel compounds for efficacy against the various stages of the Plasmodium parasite lifecycle."

Requirements of a Screening System:

The Workshop initially addressed the structure of a screening process for novel drug candidates. There was consensus that:

- Screens must be, Robust, Validated, Reproducible and Represent Human Malaria
    - *Robustness* – ensures that screens are easy to perform, low cost, suitable for screening a large variety of chemotypes or natural products
    - *Validation* – ensures that the results reflect well the activity of the compound against the targeted parasite stage
    - *Reproducibility* – Need of standardized and comparable assays for easy assessment of screening results obtained from different laboratories.
    - *Represents Human Malaria* – this may involve compromises as the use of screens using rodent and other models may be the only ones available for some time.
- Whole cell screening is a good way of identifying chemotypes with cellular permeability properties compatible with getting access to their targets inside the parasite in its various stages.
- Chemotype profiling of active compounds may provide starting points to test future hypotheses concerning drug mechanisms of action and so lead to the development of novel screens
- Once a mechanism is identified, then it should be easier to develop a screening method for that mechanism[2].

[2] This will be dealt with in Workshop No 5: "Using Chemistry to Understand Biology".

- Screening for activity against the Plasmodium parasite for the purpose of identifying potential compounds for drug development need to be viewed as two stages in a cascade that filters out inactive or inappropriate compounds:-

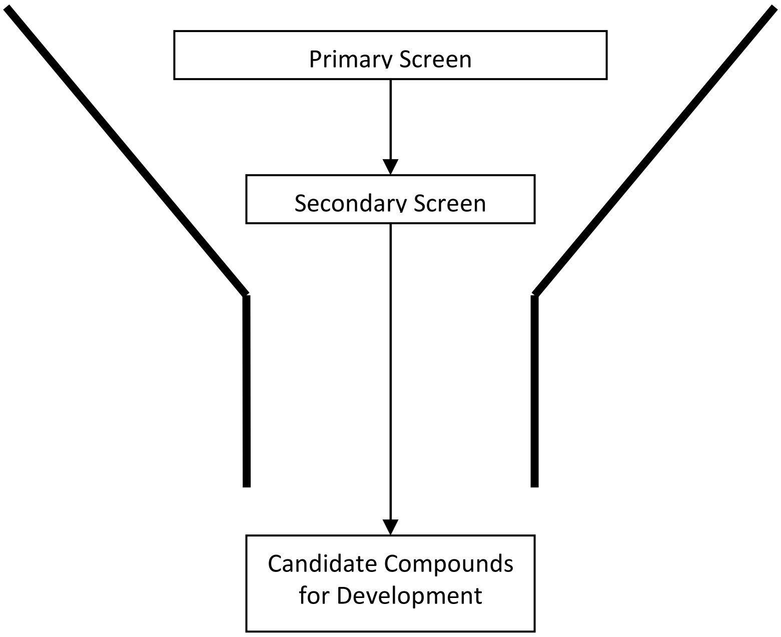

- Ideally primary screens must be simple enough to be used easily in high throughput screening systems, whereas secondary screens can be more complex and have only medium throughput capacity. However, the design of screens must not lose sight of the fact that they are to screen for activity against a particular stage of the parasite's lifecycle in the context of finding new drugs to treat malaria.
- Other methods of looking at chemotype activity that are primarily designed to determine the mode of action of a chemotype on the parasite's biology are more appropriate in later stages of the drug development process and can be more complex. They are not intended to screen for activity but to identify the mechanism of activity in depth.
- Screening systems must have built into themselves strategies to minimise the effects of false negative results.

Primary Screens:

The properties of the primary screening process should be:

- Cost-effective
- Biologically appropriate, *i.e.* the compounds actually kill the parasite
- The amount of compound needed for the screen must be small
- Very early, the screening process must exclude compounds that would be cytotoxic against the human host. This may be performed either by the choice of the library, or by using additional tests able to evaluate toxicity against host's cells.
- The screening process must include reliable models (*i.e.* new screening assays) that detect activity against **all stages** of the parasite's lifecycle. All these tests will provide a complete pharmacological profiling of all the molecules entering in the malaria portfolio.
- Possible candidate compo*unds* can either be schizonticidal *plus* activity against another stage, or only active against another stage. Purely schizonticidal drugs are probably not going to be of interest as malaria control and elimination progresses. Primary screening must take this into account.

Secondary Screens:

Secondary screening of positive hits from a primary screening system can be more biologically relevant and related to the desired activity of the target profile being pursued. They should allow translation to *in vivo* models. Secondary screening must also look for activity against drug-resistant strains of Plasmodium.

Blood Stage Screens:

P falciparum:

The existing range of screens was described as follows:-

In vitro Pharmacology (HTS Screens)	*In vivo* Pharmacology	Human Pharmacology
Radioactive (3H-Hypoxanthine)<br>Colorimetric (pLDH, HRPII)<br>Fluorescent (Syb Green)<br>Transgenic parasite (GFP, Luc reporter strains)	Human parasite models<br>• *P falciparum* in SCID mouse<br>• *Aotus/ Saimiri* NHP<br>Mouse models<br>• *P berghei* (including GFP or Luc reporter strains)<br>• *P. vinckei*<br>• *P. chabaudi*<br>• *P. yoelii*	Human Challenge

There was general agreement that this range of screens was adequate for drug screening and there was little need to prioritise additional screening methods. However several points were made:-

- Mouse models give heterogeneous results and so a variety of models need to be used in screening compounds
- Primate models are difficult, of limited availability, and not necessarily cost-effective.

Concerning severe malaria, it was clear that there is a need to identify a good *in vivo* model for that could be used in secondary screening of compounds. To find appropriate models, it is also likely that the target profile of candidate compounds need to be better defined.

P vivax:

The range of available screens for blood stage vivax activity is very limited:

In vitro Pharmacology (HTS Screens)	*In vivo* Pharmacology	Human Pharmacology
None	Human parasite models<br>• *Aotus/ Saimiri* NHP<br>Primate models<br>• *P cynomolgi*	

There is clearly a lack of *in vitro* assays that can be used in high-throughput screening systems.

The key challenge that can be identified is the lack of an *in vitro* culture system for growing *P vivax* which preferentially invades reticulocytes instead of red blood cells. At present, it is only possible to culture for one cycle *in vitro* after the parasites are taken from patients. Within this context, participants felt that the laboratory facilities being used to develop the culture method must be

beside the patients in the field. This has obvious practical issues but any need to refrigerate and transport the parasites was felt to be detrimental to the development of a reliable method.
Some workshop participants were optimistic that a standardized and long term culture of *P. vivax* parasites was not an insurmountable problem and some thought that a method could be available in the foreseeable future. However there was general agreement that this technical problem was one that needed to be properly understood if progress was going to be made in a number of related areas of vivax research.

The absence of a rodent model was seen as a roadblock to research and there was discussion about the need to prioritise the development of a SCID mouse model for vivax.

Virtually all blood stage drugs that are active against *P falciparum* are also active against *P vivax*, but the levels of sensitivity of each species to the drugs may differ. Therefore the workshop concluded that it was probably not very productive in developing primary screens that would pick up vivax activity separately. The priority should be to develop good *in vivo* models, particularly for compounds that have a differential activity against both species. Identifying mechanisms of action would also be of value but methods develop for this purpose would not be useful in screening programmes, only in determining mechanisms later in the development process.

Liver Stage Screens:

P falciparum

The range of available screens for liver stage falciparum activity is also very limited:-

In vitro Pharmacology (HTS Screens)	*In vivo* Pharmacology	Human Pharmacology
	Human parasite models<br>• SCID mouse<br>• *Aotus/ Saimiri* NHP<br>Mouse models<br>• *P berghei (GFP)*<br>• *P. vinckei*<br>• *P. chabaudi*	Human Challenge

A major priority for work on liver stages to proceed is the need to develop a reproducible and robust hepatocyte culture system for *P. falciparum* that is highly susceptible to large scale invasion by the parasite. This should ensure the complete development of the parasite and ideally give good machinery for drug metabolism. At present, only primary hepatocytes are suitable for *P falciparum* and they have very low infectivity. In addition, standardised conditions for culturing hepatocytes need to be developed. Until the scientific community has a reliable supply of liver cells to work with, it is unlikely that much can be learnt in a short space of time about the liver stages of the parasite's lifecycle. There is also a priority need to find ways of increasing the receptivity of hepatocytes to infection by sporozoites and so improve the efficiency of the screening methods that can be developed.

Another roadblock to the study and understanding of the liver stages of *P falciparum* is the need to develop a cryogenic method to store and transport sporozoites. Access to sporozoites is essential to be able to study the infective process of liver cells.

The workshop spent some time discussing if there was value in developing methods to screen for inhibitors of parasite motility. This could be of value in the development of novel drugs for prophylaxis. However it was agreed that this would be only for secondary screening and that the development of new prophylactic agents is not a priority at the moment in malaria.

P vivax:

The range of available screens for liver stage vivax activity is almost non-existent and much work needs to be done to develop appropriate screens:-

In vitro Pharmacology (HTS Screens)	*In vivo* Pharmacology	Human Pharmacology
Hepatocytes infected with *P. cynomolgi* from Rhesus monkeys	Primate models<br>• *P cynomolgi*	

The situation with screening for active liver stage activity of *P. vivax* infections is basically the same as for *P. falciparum* infections. To target *P. vivax* relapse, cellular assays for three key steps of liver-stage biology are needed: hepatocyte infection, hypnozoite formation, and reactivation to hepatic schizonts. In the absence of a biomarker, an additional challenge is how to distinguish the hypnozoites from the hepatic schizonts. The major challenge is that there is very little understanding of the biology of both the schizont stage and the long lived hypnozoite phase of the infection. The pressing question is "what is a hypnozoite, how does it work, and how can we affect its quiescent metabolism?" The workshop discussed the problems of developing an understanding of this as there is a huge heterogeneity among hypnozoites and this throws into question the practicality of developing new ways to both study and to screen compounds for activity against this dormant stage.

It was suggested that the study of mature gametocytes (Stage 5) might throw light on the hypnozoite lifecycle. Both are dormant stages of the life cycle and therefore would be expected to have areas of commonality that could be exploited. The availability and handling of Stage 5 gametocytes would be expected to be easier than hypnozoites.

It was agreed that a practical way forward (once appropriate screens have been developed) would be to screen compounds for activity against all hepatic stage activity in a primary screen and then to look at hypnozoite activity in a secondary screening process. It was suggested that in depth study of hypnozoites might be able to identify biomarkers for activity in this stage.

As with *P. falciparum*, a robust supply of *P vivax* sporozoites needs to be developed for research to proceed efficiently.

Sexual Stage Screens:

The development of drugs that are effective at blocking transmission through the sexual and mosquito-based stages of the Plasmodium lifecycle are likely to become more important as malaria control moves towards malaria elimination. The number of parasites that need to be killed in a mosquito to break the transmission cycle is orders of magnitude less than is the case in treating human infections, but this rise the question of the capacity of a drug administered to humans to affect the parasite in the mosquitoes.

Currently there is no generally accepted target profile for a drug that will act principally against the sexual stages of the parasite's lifecycle. Such a profile needs to be developed so that it is clear which stage should be targeted:-

- Gametocytes
- Oocysts
- Intermediate stages (gametes, zygotes, ookinetes)

The workshop agreed that there was no need to be screening for drugs that blocked the transition from asexual to sexual stages of the parasite as these would be hit by conventional blood schizonticides anyway. This was an interesting research topic but not of major use in novel drug discovery.

A variety of assay systems are now in development across the various sexual stages:-

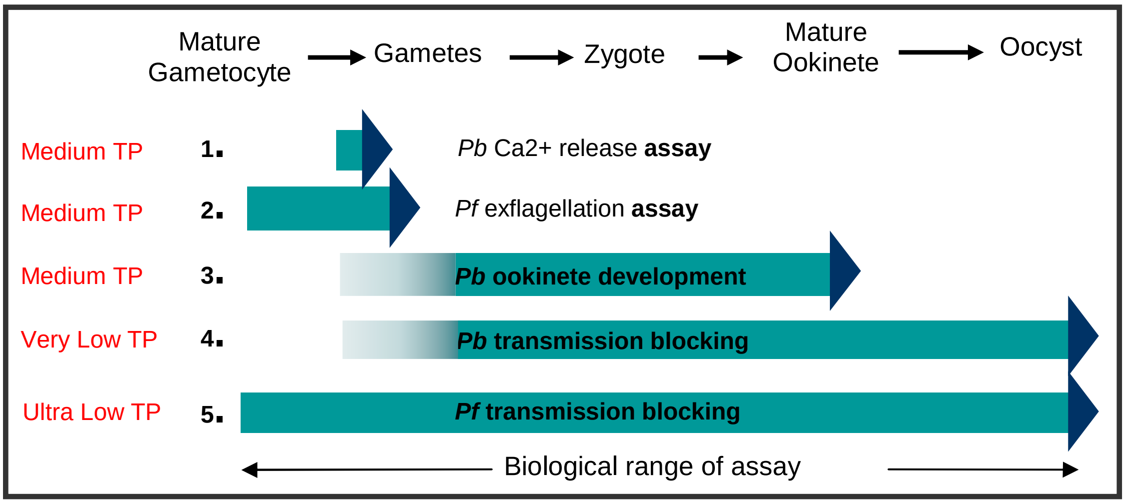

However those that can identify activity across all sexual stages (4, 5 above) have only recently been developed and currently remain low throughput systems. The *Pf* exflagellation assay is limited by the fact that it only applies to male gametocytes, but at present it is the only method to verify if Stage 5 male gametocytes are still alive.

A *P falciparum* ookinete development assay would be important for testing pharmacological activities of compounds. For this, knowledge from the development of the *P berghei* assay might be helpful. Practically a range of 2 or 3 screens distributed along the whole sexual development cycle should be used to identify potential activity in drug candidates.

The Workshop participants raise the concern about compatibility between pharmacokinetic properties of any antimalarial drugs administered to humans and the capacity to affect the late sexual stages of *. falciparum* (but NOT the other human parasites). Indeed, drugs that are active against the sexual stages of *P falciparum* will need to reside in the human body for at least 5-9 days after the appearance of symptoms in order to be present when the mature gametocytes (Stage 5) start to appear in the blood. Assuming treatment regimes remain unchanged, this has significant implications for the bioavailability and pharmacokinetic properties of drug candidates that will need to be built into the target profile. Given concerns about the development of resistance to blood schizonticides, drugs with very long half-lives, the target profile will have to address the issue of whether anti-gametocytocidal drugs should not be active against blood stages or what drug combination will be needed to protect the long half-life drug from selecting resistant strains. However consideration may be given to the design and operation of new product packaging/delivery practices that could circumvent these important issues.

Co-ordination:

It was clear from the Workshop discussions that there are many groups active in developing assays and screening methods. However there needs to be better co-ordination and networking between these groups so that there is a clear pathway for drug developers to find appropriate screens. Both MMV and TDR were suggested as possible groups to mediate and co-ordinate such a network.

Priorities for Further Work:

The Workshop identified the following areas as priorities for research work in order to speed up the discovery of novel antimalarial drugs targeted against the priority stages of the parasite lifecycle:-

<u>Blood stages</u>

- Understanding the technical problems behind the development of a *P vivax* blood stage culture system and overcoming them.
- Development of a *P vivax* blood stage rodent model.

<u>Liver stages</u>

- Development of a robust hepatocyte cell system to enable study of *P falciparum* & *P vivax* liver stages.
- Development of a standardised and improved culture system for the study of *P falciparum* & *P vivax* liver stages.
- Understanding of the nature and biology of hypnozoites with the objective of identifying a biomarker for drug activity against this stage of *P vivax* infection.
- Development of a robust and reliable supply of falciparum and vivax sporozoites and a method for safely transporting them between laboratories.
- Investigate the possibility of using a mature gametocyte and / or liver schizont assay system as a surrogate for activity against hypnozoites in primary screening for novel anti-vivax drugs.

<u>Sexual stages</u>

- Development of a target product profile for a drug to target the sexual stages of *P falciparum* and *P vivax*.
- Development of a *P falciparum* (and *P vivax*) ookinete assay to complement the current *P berghei* assay.
- Development of a gametocyte motility assay.

Next Steps:

1. Ian Boulton to draft report to be reviewed by Donatella Taramelli & Henri Vial. Then entire workshop will have an opportunity to comment before it is published on CRIMALDDI website.
2. Planned presentation as part of a CRIMALDDI Symposium at a major conference.
3. Paper written by Ian Boulton, Donatella Taramelli & Henri Vial outlining results of the workshop as part of a series of papers detailing results of the CRIMALDDI Consortium's work to be submitted for publication at end of the project. All Workshop participants who want to be included as co-authors will be added to the paper.

Ian C Boulton
01 June 2010.
