# Supplementary material for: CRIMALDDI: a prioritized research agenda to expedite the discovery of new anti-malarial drugs
Source: Malar J. 2013 Nov 5;12:395. doi: 10.1186/1475-2875-12-395 (PMC3830512; doi:10.1186/1475-2875-12-395)
Supplement: Additional file 9 — CRIMALDDI Consortium: Expert Advisory Group Meeting No. 4. [file 1475-2875-12-395-S9.pdf]

CRIMALDDI

WORKSTREAM No. 5

USING CHEMISTRY TO UNDERSTAND BIOLOGY

Report of a Workshop held at the

Liverpool School of Tropical Medicine

05 & 19 July 2010

This report is a synthesis of the face-to-face meeting on 05 July
and the subsequent videoconference on 19 July 2010.

This page is intentionally left blank

Participants:

Dr Ian Bathurst (Co-Chair)	Medicines for Malaria Venture
Prof Steve Ward (Co-Chair)	Liverpool School of Tropical Medicine
Ian Boulton (Facilitator)	TropMed Pharma Consulting
Susan Jones	Liverpool School of Tropical Medicine
Dr Neil Berry	University of Liverpool
Dr Ted Bianco	Wellcome Trust
Dr Ken Duncan	Bill & Melinda Gates Foundation (VTC)
Dr Laurent Fraisse	sanofi aventis
Dr Javier Gamo-Benito	GlaxoSmithKline
Prof Roy Goodacre	University of Manchester
Prof Neil Hall	University of Liverpool
Dr Richard Heidebrecht	Broad Institute
Prof David Hornby	University of Sheffield
Dr Alexis Nzila	KEMRI
Prof Sergio Romeo	University of Milan
Prof David Roos	University of Pennsylvania (VTC)
Prof Mike White	University of Liverpool
Prof Elizabeth Winzeler	The Scripps Research Institute (VTC)

Introduction:

Despite increasing efforts and support for antimalarial drug R&D, globally antimalarial drug discovery and development still remains largely uncoordinated and fragmented. The current window of opportunity for large scale funding of R&D into malaria is likely to narrow in the coming decade due to a contraction in available resources caused by the current economic difficulties and new priorities (*e.g.* climate change). It is therefore essential that stakeholders are given well articulated action plans and priorities to guide judgements on where their resources can be best targeted.

The CRIMALDDI[1] Consortium (a European Union funded initiative) has been set up to develop, through a structured and logical process, a focused set of detailed priorities and recommendations to address these problems. In this way it is intended to help to guide the priorities for European antimalarial research in the coming decade. It will also contribute to the wider global discovery agenda setting, and contribute to the availability of new drug candidates in the short- and medium-term.

[1] The Coordination, Rationalisation, and Integration of antiMALarial Drug Discovery & development Initiatives

The Consortium has identified 5 priority workstreams on which to focus:-

Workstream No.	Short Name	Workstream Question	Workstream Leaders
1	*Pf* & *Pv* novel targets & classes	How to identify and exploit novel targets at all stages of the lifecycle of *P falciparum* & *P vivax*.	Christian Doerig<br>Kelly Chibale
2	Managing the wealth of new HTS data	Given the large number of molecular structures that have given positive hits in the HTS screens and which are to be release by the pharmaceutical industry (>20,000), how to develop systems to:-<br>Make the information available to the community in an accessible way;<br>Filter the structures with robust methods to identify those structures which are druggable and more promising starts for lead optimisation;<br>Allow the community to know who is working on which structures so that duplication can be avoided and resources not wasted unnecessarily.	Steve Ward<br>Ian Bathurst
3	Artemisinin resistance	How to identify the mechanism(s) of artemisinin resistance in order to be able to design strategies to overcome or avoid it through novel combinations or novel molecular designs that counter the mechanism(s).	Steve Ward<br>Michael Lanzer
4	Stage-specific screening methods	How to develop a complete set of robust and replicable screening methods that can be used to screen novel compounds for efficacy against the various stages of the Plasmodium parasite lifecycle.	Donatella Taramelli<br>Henri Vial
5	Using chemistry to understand biology	How to use the results of the whole cell screening of compounds for antimalarial activity as a way of gaining insights into the underlying targets of different drug classes and then use this information to identify novel targets.	Steve Ward<br>Ian Bathurst

This is a report on the discussions and conclusions from Workshop No. 5 “Using Chemistry to Understand Biology”.

The Challenge:

Prof Steve Ward & Dr Ian Bathurst outlined the challenge that was in front of the workshop and some of the key issues that needed to be addressed.

Background:

It is anticipated that within 2010 more than 5 million chemical entities will have been screened for antimalarial activity based on whole cell screens of *P. falciparum* malaria. The screens have included fully synthetic libraries (from both commercial sources and Pharma proprietary libraries) and natural product libraries. Activity has been established based on the ability to stop the growth of asexual *P. falciparum* malaria parasites over a 24- 48hr exposure period *in vitro*. The outputs of these screens will be placed into the public domain (*e.g.* the recent announcement from GSK, one of a number of companies who have submitted their library for screening as part of an initiative driven and financed by the Medicines for Malaria Venture [MMV]).

Based on the success rates to date, there are likely to be more than 20,000 sub micromolar hits arising from these screens. The malaria genome contains about 5.5K genes of which less than 1% are likely to be druggable targets for chemotherapy. So we must conclude that many of the hits identified will be targeting the same process. In the coming year or so we expect that the stage specificity of these hits (*i.e.* activity against sexual stages and replicating and dormant liver stages) will be established, as will their *in vitro* cellular therapeutic indices, based on screens of representative mammalian cell lines.

The Challenge:

The process of taking a screening hit and progressing it through the lead identification, optimisation, and candidate selection process is expensive and time consuming. It is questionable with current resources if a significant number of these screening hits can be progressed through this pipeline without some prioritization. Knowledge of mechanisms of drug action of these hits would significantly simplify prioritization. In addition this wealth of chemical information is a fantastic resource that should be used to provide insights into the biology of the malaria parasite and the essentiality of specific processes to parasite survival.

The Workshop Goal:

By bringing together researchers with a diversity of expertise, we hope to be able to generate experimental strategies that could be used to investigate and establish the targets of the screening hits. We would wish to see what approaches would be useful in trying to group hits together that target the same parasite process in order to reduce the ≥20,000 hits to a more manageable number of molecules for lead identification. The ability to group compounds together based on a common mechanism of action should also drive basic science research into these vital parasite pathways. Furthermore, the use of the hits or leads as tools should provide new insights into the complex biology of the host-pathogen interaction at different stages of the malaria parasite life cycle.

Workshop Question:

With the situation outlined in the Challenge section, the workshop was asked to address the following question:-

- "Can we use the output of the whole cell screening of compounds for antimalarial activity as a way to identify targets of different drug classes and then use this information and the compounds as tools to gain insights into the underlying biological mechanisms of action?"

As will be seen later in the report, the participants, following discussion, changed the focus of the Question to be answered.

Boundaries of the Discussion:

The Workshop was designed to be complementary to Workshop No 2 ("Managing the wealth of new HTS data" – see above) and to develop the discussion on what is the most efficient way to take the information from the recently published positive hits in HTS results co-ordinated by MMV[2,3] and use this to identify and progress the most promising potential compounds in drug discovery and development. Given the attrition rate of drug development and the potential resources that can be assigned to antimalarial drug development, it is likely that the 20,000 hits will only result in 2-3 actual drugs reaching the patients. The Workshop participants agreed that the discussion should focus on:

- Blood stage *P falciparum* activity (this was what the HTS campaigns were screening for)[4].
- Approximately 20,000 positive hits recently published.
- Priority is to develop new drugs meeting the published target profiles.

Participants' Initial Response to the Challenge & Question

It is clear that the process of looking for new drugs and new drug targets can be divided into 3 possible approaches:-

1. Familiar chemistry applied to novel target(s);
2. Familiar targets and identifying novel chemistry;
3. Identifying a hypothetical target and screening for chemistry active against this ("fishing for new chemistry")

This particular workshop focussed on taking the chemical structures that have been shown to kill *P falciparum* in red blood cells in vitro, and from these results try and identify novel targets from a better understanding of the underlying chemistry and parasite biology. It was agreed that, in finding novel drug candidates most efficiently, then the 20,000 positive hits from the HTS data needed to be collapsed *via* secondary screening strategies to about 200 compounds. Identifying ways of achieving this was the one of the subjects of Workshop 4 ("Stage Specific Screening Methods"). Once the number of structures has been reduced to this more manageable number, from a purely drug discovery perspective, then it makes more sense to seek the targets for the structures and understand the mode of action.

The whole cell screening approach reflects the actual situation that a drug faces with the parasite, but the lack of an understanding of the actual target and mode of action means that lead optimisation is difficult. This is especially true if a particular drug may be active against several targets in the parasite. More use of *in silico* techniques to cluster compounds with common structural features and relate them to hypothetical pharmacophores could be an approach. However to-date such analysis in malaria has not been very promising. At least one participant felt that more resources should be put into this area as it has shown promise in other fields.

In other areas, the combination of quantitative measures coupled with appropriate mathematical models has been very useful in working out biological mechanisms. The models can be developed from the knowledge of the activity of a particular drug and modes of action can be hypothesised and then modelled. However the participants working on malaria were concerned that chemoinformatics for this disease needed a lot more work. Access to data is a rate-limiting step and is on the critical path to successful target & lead identification.

---

[2] Gamo FJ, Sanz LM, Vidal J, de Cozar C, Alvarez E, Lavandera JL, *et al.*. Thousands of chemical starting points for antimalarial lead identification. Nature 2010; 465 (7296): 305-310.
[3] Guiguemde WA, Shelat AA, Bouck D, Duffy S, Crowther GJ, Davis PH, *et al.*. Chemical genetics of *Plasmodium falciparum*. Nature 2010; 465 (7296): 311-315.
[4] The Genomics Institute of the Novartis Research Foundation reported that data on the liver and activity of these compounds should be available shortly.

The Workshop participants were concerned that the screening results could be misleading for two main reasons:-

- The effect of the compound may not be on the parasite but on the red blood cell (whether infected or not);
- The compounds used in the screen may not be adequately pure and the instability has resulted in contaminated samples, leading to incorrect interpretation of the compounds activity.

It is well known that screens against known targets do not always correlate to the results of whole cell screens for the same compounds. This may indicate that the effect of compounds on the parasite is more complex than activity simply against a single protein target. It may be that large protein assemblies are involved in drug activity rather than just simple single protein targets.

Studying the genomic response to chemical compounds is a promising approach to elucidate mechanism of action. Phenotypic screens can be used for cluster analyses to identify molecules with similar modes of action. An understanding of the relationship between resistance to particular classes of compounds and the underlying genomic changes leading to the resistance may be able to shed some light on the underlying biology of a compound class' activity. On the other hand, a single newly-emerged mutation may be detected using full-genome microarrays. This approach is substantially less expensive to implement than sequencing and allows for the analysis of more samples but with a possibly higher false negative rate. Cost is an issue because the analysis of multiple independent resistant genomes will be critical for selecting the true target from the five-ten possible targets revealed by each selection. The arrays have an added advantage in that the bioinformatics analysis is relatively straightforward. A bottleneck in the approach is confirming mutations by molecular biology because genetic manipulation (especially transformations) in *P falciparum* is not easy. However this approach is probably the most promising in the short-term.

Other possible approaches were suggested and discussed:-

- *Metabolomics:* may be informative but much more work is needed on the malaria parasite metabolome before this approach is likely to be useful. The main problem is that it is not clear where in the metabolome to look for drug-induced perturbations. The timing of the effect of the compound is important – rapid kill being a key element of the target profile – but developing a screen at scale to look at this is a challenge.
- *Proteomics:* currently profiles identified have not proven to be very useful in elucidating mechanisms of action or suggesting novel targets.
- *Transcriptomics:* has been very useful in other fields such as TB, where compounds can be clustered according to their general mode of action (acting on transcription, translation, *etc*.) or identified as having a novel mechanism. However, the technology does not currently allow one to deduce the mode of action. Like proteomics, for malaria the current profiles have not yet been very useful, especially for compounds with unknown mechanisms of action.

There was a feeling among those working on malaria in the meetings that to-date there are few examples of finding targets or understanding modes of action without a pretty good idea ahead of time of what one was looking for. However in TB investment in generating a suite of tools has paid off and there is a good chance that good drug targets will be identified starting from whole cell screening hits.

HTS data is a valuable starting point for identifying novel compounds, but the process has huge error bars around the results and there is a risk of over-interpreting negative results. Positive hits need to be reconfirmed in several screens before committing to lead optimisation and understanding modes of action.

The use of surrogates for human malarial infections was discussed as ways of better understanding modes of action and identifying putative targets. Rodent models have been popular for decades and still have a valuable role despite the development of humanised mouse models (which remain expensive). The use of Toxoplasma screens was also recommended, but this was felt by some participants to be too restricted, looking for example at novel folate inhibitors rather *etc* rather than finding totally novel ways of attacking the parasite. The majority of compounds with blood stage activity do not appear to have potent activity against *T gondii*.

Currently MMV reacts to proposals submitted to it for funding within the overall drug R&D strategic plan. However there was a feeling from the Workshop that MMV may need to be more proactive in driving the agenda on some aspects of drug discovery in order to ensure that the right approaches, compound classes, and targets were prioritised and actively pursued. As is noted below, there was a call from the Workshop for MMV to play a greater role in co-ordinating and communicating the work being done in this area. It was noted that the funding agencies respond better to a clear and aligned message on priorities of needs and MMV is probably best placed to ensure this alignment. Also the availability of adequate quantities of compounds to undertake experiments was identified as a significant challenge. The availability of adequate amounts of compounds for further work is also on the critical path to success in finding new drugs and new targets There needs to be one organisation co-ordinating this and again MMV was identified as being in the best position to do this.

Revised Question:

The Workshop participants agreed that the Workshop Question could be better formulated as follows:-

> "What are the Strategies and Tools that should be prioritised in antimalarial drug discovery in order to address two key challenges:-
>
> - Identify and take forward in discovery & development as quickly as possible novel drug compound classes;
> - Understand the underlying biology of the activity of compounds identified as being active against *P falciparum* to inform the choice of novel chemical structures to address unmet need in antimalarial drugs."

Identifying Novel Drug Candidates as Quickly as Possible:

The Workshop participants identified the following as priority needs to move forward the search for novel drug candidates working from the information contained in the 20,000 positive hits now in the public domain:-

Priority	Need
One	• Understanding of the ADME and toxicological properties of the positive hits and the flexibility to address any shortfalls in these through structural manipulation during lead identification and optimisation.<br>• Increased resources devoted to classic medicinal chemistry to synthesise the compounds needed to address the bullet point above and also to yield adequate amounts of compounds to undertake more in-depth examination of the compounds activities.<br>• Phenotyping of the parasite strains that are affected by different chemical classes in order to yield information on the targets in the genome for each class.<br>• Use of Information Technology tools to identify clustering of chemical structures around particular biological activities that can then be used to design drugs with properties more closely aligned to those needed for practical drugs.
Two	• Increased work to validate targets to ensure that active compounds are active on targets that are essential to the parasite's biology.<br>• Fill in the gaps in tools and reagents needed to meet the needs of this area. Areas for investment in new tools include the use of affinity tags and antibodies. The development of an affordable humanised mouse model was also identified as a priority.<br>• Tools such as imaging of the parasite's response, metabolomics, transcriptomics, proteomics, structural algorithms, and resistant parasite lines are already available. However, with the exception of work on resistant lines, to-date the application of these tools has not given very promising results and more work needs to be done before they are considered to be useful in malaria R&D.
Three	• Increased and improved co-ordination and communication of what activity is being undertaken, who is undertaking it, and what are the results they are achieving.[5] MMV was seen as the obvious organisation to lead this.

[5] See the recommendations from Workshop 2 on co-ordination and communication relating to the availability of the 20,000 hits recently published.

Understanding the Underlying Biology:

The Workshop envisaged that efforts to understand the underlying biology from the available information on active chemical structures should be undertaken in parallel to the work on quickly identifying the most promising compound classes to pursue as new drugs. The priorities for work in this area were agreed to be:-

Priority	Need
One	• Use of knowledge of the parasite's resistance to different chemical classes to probe (through genomics and other tools) the underlying biology.<br>• Phenotyping of the parasite strains that are affected by different chemical classes in order to yield information on the targets in the genome for each class.
Two	• Database design and curation to ensure that the information on structural relations to activity can be easily accessed (a recommendation of Workshop 2).<br>• Increased resources devoted to ensuring the availability of adequate quantities of active compounds for proper experiments on activity and targets.<br>• Fill in the gaps in tools and reagents needed to meet the needs of this area. Areas for investment in new tools include the use of affinity tags and antibodies, genetic crosses between strains and species of Plasmodium. The development of an affordable humanised mouse model was also identified as a priority.<br>• Improvements in molecular biology and elevation of this beyond antimalarial drug discovery to an understanding of malaria in general.<br>• Tools such as imaging of the parasite's response, metabolomics, transcriptomics, proteomics, structural algorithms, as well as tools for looking at resistant parasite lines on a genomic scale are already available. The challenge is the practical one of being able to create resistant lines for each compound or class of compounds. However, with the exception of work on creating and analysing resistant lines, to-date the application of these tools has not given very promising results and more work needs to be done before they are considered to be useful in malaria R&D.<br>• Appropriate mathematical models of biological pathways are available but access to adequate amounts and quality of data to use with them is still difficult.<br>• Imaging tools for the mechanisms of parasite drug resistance and mode of action are available but need improving.<br>• Development of models and modelling methods to allow theories about parasite biology and drug targets to be investigated and experiments designed to test modelling results.
Three	• Increased and improved co-ordination and communication of what activity is being undertaken, who is undertaking it, and what are the results they are achieving.[6] MMV was seen as the obvious organisation to lead this.<br>• Use of Information Technology tools to identify clustering of targets around particular chemical structural classes that can then be used to identify common aspects of the targets and / or compound structures that can inform theories about targets & parasite biology.<br>• Increasing the chemical diversity of active compounds in order to widen the information inputs to the consideration of biological mechanisms.

[6] See the recommendations from Workshop 2 on co-ordination and communication relating to the availability of the 20,000 hits recently published.

Next Steps:

1. Ian Boulton to draft report to be reviewed by Steve Ward & Ian Bathurst. Then entire workshop will have an opportunity to comment before it is published on CRIMALDDI website.
2. Planned presentation as part of a CRIMALDDI Symposium at a major conference.
3. Paper written by Ian Boulton, Steve Ward, & Ian Bathurst outlining results of the workshop as part of a series of papers detailing results of the CRIMALDDI Consortium's work to be submitted for publication at end of the project. All Workshop participants who want to be included as co-authors will be added to the paper.

Ian C Boulton
04 August 2010.
